# Engineering ROS‐Responsive Bioscaffolds for Disrupting Myeloid Cell‐Driven Immunosuppressive Niche to Enhance PD‐L1 Blockade‐Based Postablative Immunotherapy

**DOI:** 10.1002/advs.202104619

**Published:** 2022-02-13

**Authors:** Shaoyue Li, Chunyan Zhu, Xianli Zhou, Liang Chen, Xiaowan Bo, Yuting Shen, Xin Guan, Xiaoxia Han, Dandan Shan, Liping Sun, Yu Chen, Huixiong Xu, Wenwen Yue

**Affiliations:** ^1^ Department of Medical Ultrasound Shanghai Tenth People's Hospital Ultrasound Research and Education Institute School of Medicine Tongji University Shanghai Engineering Research Center of Ultrasound Diagnosis and Treatment National Clinical Research Center of Interventional Medicine Shanghai 200072 P. R. China; ^2^ Department of In‐patient Ultrasound The Second Affiliated Hospital Harbin Medical University Harbin 150001 P. R. China; ^3^ Department of Gastroenterology Shanghai Tenth People's Hospital Tongji University School of Medicine Shanghai 200072 P. R. China; ^4^ Materdicine Lab School of Life Sciences Shanghai University Shanghai 200444 P. R. China; ^5^ Department of Ultrasound Zhongshan Hospital Fudan University Shanghai 200032 P. R. China

**Keywords:** checkpoint blockade, immunotherapy, myeloid cells, nanomedicine, tumor therapy

## Abstract

The existence of inadequate ablation remains an important cause of treatment failure for loco‐regional ablation therapies. Here, using a preclinical model, it is reported that inadequate microwave ablation (iMWA) induces immunosuppressive niche predominated by myeloid cells. The gene signature of ablated tumor presented by transcriptome analyses is highly correlated with immune checkpoint blocking (ICB) resistance. Thus, an in situ scaffold with synergistic delivery of IPI549 and anti‐programmed death‐ligand 1 blocking antibody (aPDL1) for postablative cancer immunotherapy is designed and engineered, in which IPI549 capable of targeting myeloid cells could disrupt the immunosuppressive niche and subsequently improve ICB‐mediated antitumor immune response. Based on five mouse cancer models, it is demonstrated that this biomaterial system (aPDL1&IPI549@Gel) could mimic a “hot” tumor‐immunity niche to inhibit tumor progression and metastasis, and protect cured mice against tumor rechallenge. This work enables a new standard‐of‐care paradigm for the immunotherapy of myeloid cells‐mediated “cold” tumors after loco‐regional inadequate practices.

## Graphical abstract

The in situ scaffold enables local retention and controlled release of therapeutics (anti‐programmed death‐ligand 1 blocking antibody (aPDL1) and IPI549) for postablative cancer immunotherapy and mimics a “hot” tumor‐immunity niche, in which IPI549 capable of targeting myeloid cells could disrupt the immunosuppressive niche and subsequently improve immune checkpoint blocking‐mediated antitumor immune response.

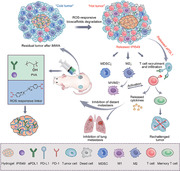

## 1 Introduction

Compelling clinical findings in cancer immunotherapy have opened up a new era of tumor treatment,^[^ ^1^, ^2^ ^]^ and the anti‐programmed death‐1(PD‐1)/programmed death‐ligand 1 (PD‐L1) agents have been recognized as the backbone of those progressively expanding strategies in the immune‐oncology field.^[^ ^3^ ^]^ However, considering the complexity of anticancer immunity, several approaches targeting multiple pathways should be combined to acquire effective systemic therapies.^[^ ^4^ ^]^ Among them, determining the best way to integrate immune checkpoint inhibitors with loco‐regional percutaneous thermal ablation (PTA) therapies (e.g., radiofrequency ablation (RFA), microwave ablation (MWA), laser ablation (LA)) remains an extremely active area of scientific investigation, mainly owing to the central role of PTA in curative treatment of a wide array of malignancies (such as lung, liver, thyroid cancers and colon cancer metastases) supported by extensive worldwide experience^[^ ^5^, ^6^, ^7^, ^8^ ^]^ and even international clinical guidelines.^[^ ^9^, ^10^ ^]^ Furthermore, PTA can liberate an abundance of tumor‐associated antigens and certain “danger” signals,^[^ ^11^ ^]^ lending itself to favorably combine with existing immunotherapeutic strategies. However, despite the presence of some advantages, the high rate (60–80% post local ablation) of lesion recurrence or inadequate ablation remains a therapeutic dilemma for any loco‐regional PTA treatment.^[^ ^12^, ^13^ ^]^ Importantly, evidence highlights that the altered tumor microenvironment (TME) post‐ablation stimulates growth of residual tumor, which harbors an aggressive phenotype and an unfavorable prognosis.^[^ ^13^ ^]^ Although the fact that cancer metastasis frequently arising at the site of injury has been well recognized for over a century,^[^ ^14^ ^]^ the specific mechanisms that link local ablation‐induced tissue trauma to the rapid growth of residual tumor are still poorly understood. As such, strategies to reveal and inhibit the underlying principles responsible for such pro‐oncogenic effects should be crucial to maximizing the clinical response of PTA‐based antitumor immunotherapies.

The tumor‐associated myeloid cells (TAMCs) originating from hematopoietic precursors are a heterogeneous population mainly including myeloid‐derived suppressor cells (MDSCs) and tumor‐associated macrophages (TAMs).^[^ ^15^ ^]^ Although phenotypically and morphologically distinct, they share a functional commonality that dramatically impairs both innate and adaptive antitumor immunity.^[^ ^16^ ^]^ In addition, the growing evidence implies that high infiltration of TAMCs also correlates with the poor prognosis and particularly the resistance to immune checkpoint blocking (ICB).^[^ ^17^ ^]^ Here in this work, with a preclinical subcutaneous implantation murine model of colon adenocarcinoma, we demonstrate that rapid tumor progression induced by inadequate MWA (iMWA) is dominantly mediated by the so‐called “cold” tumor immune milieu, which is characterized by the enrichment of immune suppressors (e.g., MDSCs, TAMs) together with a paucity of cytotoxic T‐lymphocyte (CTL) infiltration. Relatedly, the gene signature of post‐iMWA wound‐healing presented by transcriptome analyses is highly correlated with ICB resistance in clinic. Previous studies^[^ ^18^ ^]^ have proved that the gamma isoform of phosphoinositide 3‐kinase (PI3K*γ*) is highly expressed in myeloid suppressor cells and contributed to promoting migration and production of various inflammatory mediators. Also, mice lacking p110*γ*, a subunit of PI3K*γ*, show reduced immunosuppressive TAMCs and tumor growth.^[^ ^19^ ^]^ Thus, therapeutics actively targeting TAMCs via PI3K*γ* blockade can be rationally employed to modulate the TME of residual tumor post‐ablation in hopes of maximizing therapeutic index of ICB‐based therapies following PTA.

**Figure 1:**
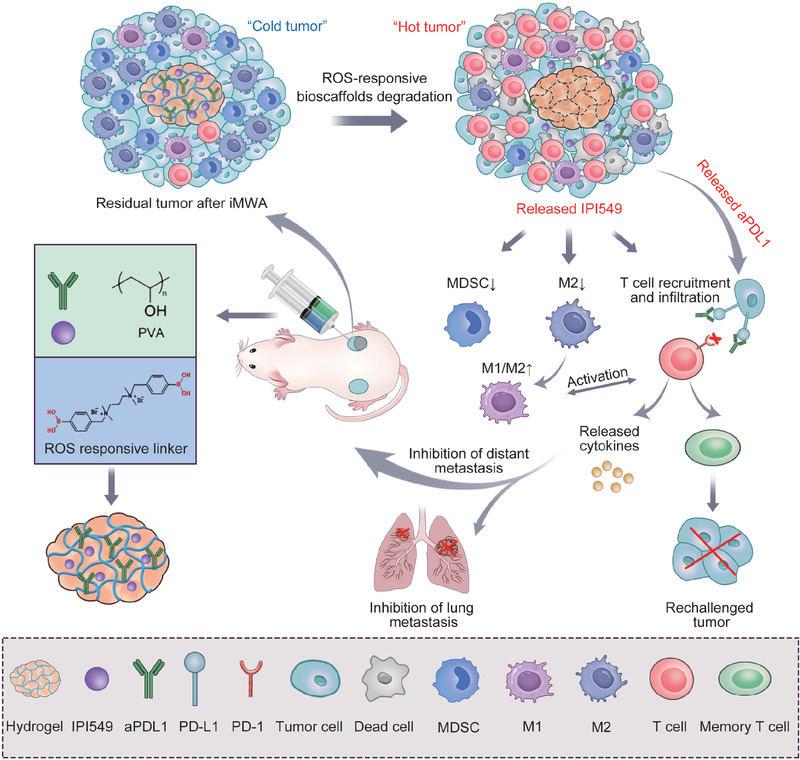
Schematic of engineering ROS‐responsive bioscaffolds disrupting myeloid cell‐driven immunosuppressive niche to enhance PD‐L1 blockade‐based postablative immunotherapy. Inadequate microwave ablation (iMWA) induces immunosuppressive niche predominated by myeloid cells. The in situ gelation involved in this strategy enables local retention and controlled release of therapeutics (aPDL1 and IPI549), in which IPI549 capable of targeting myeloid cells‐induced immunosuppression and subsequently improving PD‐L1 blockade‐mediated antitumor immune response. This biomaterial system (aPDL1&IPI549@Gel) mimics a “hot” tumor‐immunity niche to inhibit tumor progression and metastasis, and protect cured mice against tumor rechallenge. ROS, reactive oxygen species; aPDL1, anti‐programmed death‐ligand 1 blocking antibody; PD‐L1, programmed death‐ligand 1; PD‐1, programmed death‐1; MDSC, myeloid‐derived suppressor cell; M1, M1‐like macrophage; M2, M2‐like macrophage.

Rapid expansion in the bioengineering and nanotechnology fields affords new approaches^[^ ^20^, ^21^, ^22^, ^23^ ^]^ that could dramatically improve the safety as well as the therapeutic effectiveness of cancer immunotherapy by enabling efficient immunomodulatory compounds delivery.^[^ ^24^, ^25^, ^26^, ^27^ ^]^ Notably, given that systemic administration of immunotherapeutic agents would disrupt the homeostatic function of immune cells at the non‐target tissues,^[^ ^28^ ^]^ the localized delivery vehicles can allow regulated and sustained release of payloads, thereby not only minimizing the off‐target associated side effects but also enhancing efficient drug bioavailability.^[^ ^29^ ^]^ Based on the above analysis, we herein propose an injectable reactive oxygen species (ROS)‐sensitive in situ hydrogel scaffold for locally synergistic delivery of a selective pharmacological PI3K*γ* inhibitor (IPI549) and an anti‐PD‐L1 blocking antibody (aPDL1) (designated as aPDL1&IPI549@Gel) to maximize the anticancer efficacy, in which PI3K*γ* inhibition should likely reverse the immunosuppressive niche after iMWA and subsequently promote ICB‐mediated antitumor immune responses (**Figure** **1**). We demonstrated that IPI549‐loaded hydrogel could reshape the tumor immune microenvironment (TIME) by reducing CD11b^+^ suppressive immune cells including MDSCs and TAMs, and also enhancing infiltration of CD8^+^ T cells. Together with the sustained release of aPDL1, the ROS‐responsive depot could elicit strong anticancer immune responses, which imitated a “hot” tumor immunity niche, regressed/eradicated primary tumor following ablation, inhibited the development of distant and spreading metastasis, as well as provided strong long‐term immunological memory protection for treated mice. Given the clinical central role of PTA represented by MWA for a wide array of patients with solid tumors, our proposed post‐ablation immunotherapy approach holds considerable potential to enable a new standard‐of‐care paradigm in the interventional oncology.

## 2 Results and Discussion

### 2.1 iMWA Promotes Tumor Progression and Induces Immune Suppression Mediated by Myeloid Cells

**Figure 2:**
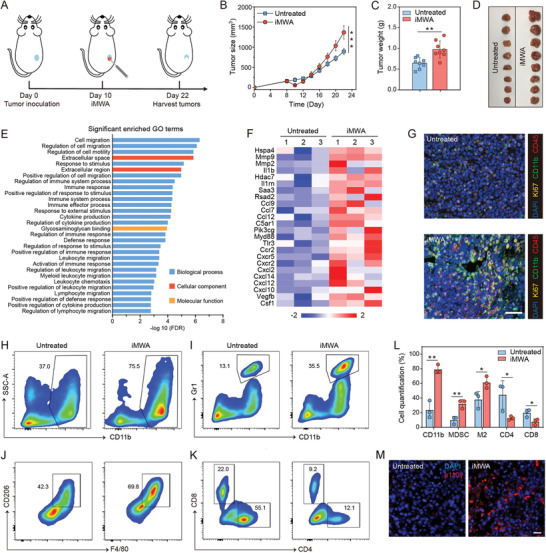
iMWA promoting tumor progression and inducing immune suppression. A) Schematic illustration of inadequate microwave ablation (iMWA) treatment. B) Residual tumor growth kinetics and C) residual tumor weight of untreated and iMWA mice on day 22. Data are given as means ± standard deviation (SD) (*n* = 8). D) Photographs of tumors on day 22 in two groups. E) Significant enrichment in gene ontology (GO) terms. The *y*–axis shows GO categories, and the *x*–axis refers to the enrichment false discovery rate (FDR). F) Heat map of mean fold‐change in gene expression of chemokines and immune suppression (*n* = 3). G) Representative immunofluorescence images of residual tumors showing CD45^+^ cells, CD11b^+^ cells, and Ki67 infiltration. Scale bar: 25 µm. H) Representative flow cytometric analysis of CD11b^+^ cells and I) myeloid‐derived suppressor cells (MDSCs; CD11b^+^Gr1^+^) gating on CD45^+^ cells. J) Representative flow cytometric analysis of M2‐like macrophages (F4/80^+^CD206^hi^) gating on CD45^+^CD11b^+^ cells. K) Representative flow cytometric analysis of CD8^+^ and CD4^+^ T cells gating on CD45^+^CD3^+^ cells. L) Flow cytometric quantification of the above cells. Data are presented as means ± SD (n  =  3). M) Representative immunofluorescence images of tumors showing p110*γ* (red) expression for untreated and iMWA groups. Tissues were counterstained with DAPI (blue) to detect nuclei. Scale bar: 20 µm. Statistical significance was calculated by Student's *t* test. *p < 0.05, **p < 0.01 and ***p < 0.001.

To investigate whether inadequate ablation could induce tumor progression, a preclinical colon adenocarcinoma murine model which is an immunocompetent host was introduced into this study. iMWA was performed on the tumor‐bearing mice after inoculating CT26 cancer cells for 10 days (**Figure** **2**A). Afterward, tumor burden was monitored by bioluminescence imaging and caliper measurement (Figure S1, Supporting Information). We found that despite iMWA initially reducing the size of the treated tumors, the residual tumors post‐ablation eventually had a larger growth slope as compared to the untreated ones (Figure 2B). Furthermore, on day 22 after treatment, the tumors were harvested. As indicated in Figure 2C,D, the variation trend of tumor weight was consistent with that of tumor volume. These results confirmed that local ablation‐induced tissue injury could promote the rapid outgrowth of residual tumors.

To further explore the underlying mechanism of the promoted tumor progression effects of iMWA, we conducted RNA‐seq analysis for the residual CT26 tumors 3 days after iMWA as well as untreated controls. As expected, the results presented that the gene expression profiles of iMWA‐treated tumors were obviously distinct from the control ones, with a total of 3452 differentially expressed gene profiles between two groups (Figure S2, Supporting Information). Furthermore, gene ontology (GO) analysis was performed, including biological process, cellular component, and molecular function immune (Figure 2E). Among them, the top enriched terms included immune response, immune system process, leukocyte chemotaxis, and myeloid leukocyte migration. Also, genes encoding proinflammatory cytokines, chemokines, and damage‐associated molecular patterns molecules (DAMPs), as well as immunosuppression‐related genes were significantly overexpressed in the ablated tumors (Figure 2F), which suggested that the immune microenvironment in the residual tumor post‐iMWA had changed. Relatedly, the genes which are highly correlated with ICB resistance in clinic^[^ ^30^ ^]^ were expressed higher among post‐iMWA tumor tissues relative to untreated ones (Figure S3, Supporting Information). These data indicated that iMWA triggered complex inflammatory reactions in residual tumors, which ultimately promoted the generation of TIME and resistance to ICB therapy.

During inflammation responses, monocytes and granulocytes from peripheral blood can be recruited into injured tissues.^[^ ^31^, ^32^ ^]^ These cells express inflammatory factors that play a vital role in recruiting additional immune cells and particularly the clusters of myeloid cells (CD11b^+^) to the site of damage.^[^ ^33^ ^]^ Growing evidence has shown that TAMCs correlate with immunosuppressive, ICB resistance, and even poor prognosis.^[^ ^18^, ^19^ ^]^ Therefore, we analyzed the infiltrating immune cellular phenotype of residual tumors after local ablation. 3 days after iMWA, the results of multiple immunohistochemistry (mIHC) assay demonstrated that the infiltration of CD45^+^ cells in residual tumor was significantly enhanced (Figure 2G). Notably, the CD11b^+^ myeloid cells were increased in comparison to that in untreated control. Also, we found that the proliferative index (displayed by Ki67 staining) of ablated tumor was much higher than that of non‐treated tissue (Figure 2G). Besides, it was found that PD‐L1 also presented higher expression in the residual tumor post‐iMWA (Figure S4, Supporting Information), highly indicating the pro‐tumorigenic effects of inadequate ablation‐induced inflammatory TIME.

In order to further assess the cellular mechanisms of such an immunosuppressive effect, flow cytometric analysis was conducted for the fresh residual CT26 tumor tissues 3 days after ablation. Consistent with results of mIHC, iMWA strongly promoted the accumulation of CD11b^+^ myeloid cells (Figure 2H). We further subdivided these myeloid cells into MDSCs (CD11b^+^Gr1^+^), M1‐like macrophages (CD11b^+^F4/80^+^CD80^hi^, TAMs‐M1) and M2‐like macrophages (CD11b^+^F4/80^+^CD206^hi^, TAMs‐M2).^[^ ^34^ ^]^ As is known, infiltration of TAMs‐M1 is associated with good prognosis for cancer patients, while TAMs‐M2 participate in tissue remodeling and immune regulation, even highly express immunosuppressive molecules and enable formation of immune escape.^[^ ^35^ ^]^ Hence, TAMs‐M2 correlate with tumor cell proliferation and worse clinical outcomes.^[^ ^34^ ^]^ Interestingly, both MDSCs and TAMs‐M2 showed a substantial increase in the residual tumors as compared to the untreated ones on day 3 after treatment (Figure 2I,J). Notably, we also found a significant suppression of CD8^+^ T cells and CD4^+^ T cells (Figure 2K,L), which corresponded to previous reports of the T cell suppression effect induced by TAMCs.^[^ ^18^, ^34^ ^]^ Taken together, the above findings suggested that inadequate ablation could induce a “cold” tumor immune milieu, which was characterized by the increased influx of myeloid cells and a paucity of CTL infiltration, thus eventually promoting tumor progression.

### 2.2 PI3K*γ* as the Research Target of Inadequate Ablation

In order to efficiently eliminate immunosuppression and reactivate the immune response after iMWA, PI3K*γ*, one of the key proteins associated with immune suppression,^[^ ^18^ ^]^ serves as a potential therapeutic target for post‐ablation treatment. PI3K*γ* is the most highly expressed PI3K isoform in myeloid cells and can accelerate the production and recruitment of inflammatory factors.^[^ ^18^, ^34^ ^]^ Importantly, PI3K*γ* works downstream of diverse chemoattractant–receptor pairs, such as CC chemokine receptor 2 (CCR2), interleukin 1 receptor (IL‐1R), and vascular endothelial growth factor receptor 1 (VEGFR1), to promote the migration of myeloid cells to tumors.^[^ ^36^ ^]^ These studies prompt that targeting myeloid suppressor cells trafficking to tumors by blocking PI3K*γ*‐mediated signaling pathway should likely represent an effective way of inhibiting tumor immunosuppression and improving antitumor immunity. Especially, in this work, gene expressions of PI3K*γ* and its related chemoattractants were upregulated based on results of RNA‐seq analysis (Figure 2F). Immunostaining for PI3K*γ* also showed an enhanced intensity in ablated tumor compared with control group (Figure 2M). Considering the abovementioned gene signature of ICB resistance, therapeutics actively targeting PI3K*γ* can be rationally utilized to maximize the subversion of tumor immunosuppression caused by myeloid cells infiltration and subsequently improve therapeutic index of ICB immunotherapy following inadequate ablation.

### 2.3 Synthesis and Characterization of the Bioresponsive Scaffold

**Figure 3:**
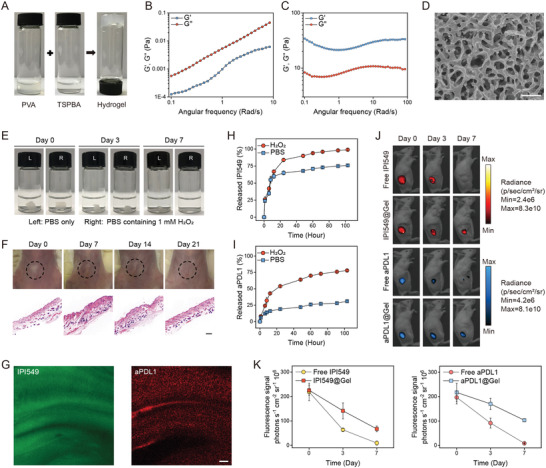
Synthesis and characterization of in situ formed bioresponsive scaffold. A) Photographs of hydrogel formation. B) Frequency dependency of the elastic (G′) and viscous (G″) moduli of PVA and C) PVA‐TSPBA hydrogel samples. D) Cryo–scanning electron microscopy (Cyro‐SEM) image of hydrogel. Scale bar: 1 µm. E) ROS‐sensitive gel in PBS and H_2_O_2_ solution. F) Images of skins at the sites of injecting hydrogel and their corresponding hematoxylin and eosin (H&E) staining results at different time points. Black circles indicate hydrogel. Scale bar: 250 µm. G) Representative fluorescence images of the hydrogel in which fluorescein isothiocyanate (FITC) (green) was used as the substitution of IPI549 and anti‐programmed death‐ligand 1 blocking antibody (aPDL1) labeled with Cy5.5 (red) was used as the substitution of aPDL1. Scale bar: 5 µm. H) Cumulative release profiles of IPI549 and I) aPDL1 from hydrogel incubated in PBS with or without 1 mm H_2_O_2_. Data are presented as means ± SD (*n* = 3). J) In vivo retention of IPI549 and aPDL1 in different formulations at different days (days 0, 3, and 7) injected subcutaneously into CT26 tumor‐bearing BALB/c mice and K) their corresponding quantitative analysis of fluorescence signals. Data are presented as means ± SD (*n* = 3).

A vast scale of delivery systems based on nanomedicine has been well‐demonstrated to enable efficient antitumor immunotherapy compared with free immunotherapeutic administration. Among them, hydrogel‐based drug‐delivery systems, especially stimuli‐responsive ones, have recently received tremendous attention due to their strong altered‐drug efficacy such as tissue redistribution, enabling sustained and controlled release of therapeutic agents.^[^ ^37^ ^]^ Interestingly, we found that inadequate ablation could increase the formation of ROS in the residual tumor (Figure S5, Supporting Information). Previous study shows that inadequate ablation can enhance blood flow in the sublethal areas, which results in increased oxygenation that can contribute to the formation of ROS.^[^ ^31^ ^]^ Encouraged by the above findings, a ROS‐responsive degradable scaffold was introduced in this study (**Figure** **3**A), which was formed by combining cross‐linked poly (vinyl alcohol) (PVA) and N^1^‐(4‐boronobenzyl)‐N^3^‐(4‐boronophenyl)‐N^1^,N^1^,N^3^,N^3^‐tetramethylpropane‐1,3‐diaminium (TSPBA) with a volume ratio of 1:1. The ROS‐labile TSPBA linker was synthesized using quaternization reaction of N^1^,N^1^,N^3^,N^3^‐tetramethylpropane‐1,3‐diamine with excess 4‐(bromomethyl) phenylboronic acid, and the results of ^1^H‐NMR showed the successful synthesis of TSPBA and demonstrated it could be hydrolyzed when exposed to H_2_O_2_ (Figure S6, Supporting Information). The character of gel was tested by a rheology analysis including elastic (G′) and viscous (G″) moduli. As expected, G' was rapidly increased with TSPBA addition to aqueous solution of PVA, proving that a network was formed among the PVA chains (Figure 3B,C). The porous network microstructure of this bioresponsive scaffold was obtained by cryo–scanning electron microscopy (Cryo‐SEM) analysis (Figure 3D). The in vitro degradation of ROS‐sensitive hydrogel was evaluated in the presence of phosphate‐buffered saline (PBS) with H_2_O_2_ at 37 °C. Compared with that in PBS solution, the gel exposed in H_2_O_2_ solution degraded much faster (Figure 3E and Figure S7, Supporting Information). The biodegradability and biocompatibility of the obtained gel were further texted in vivo by utilizing healthy BALB/c mice. As displayed in Figure 3F, the overall size of gel decreased gradually at the injection site over time, and on the 21st day after injection, the gel completely disappeared, and there was no appreciable skin inflammatory effect as shown in hematoxylin and eosin (H&E) staining test.

Next, we evaluated the ability of loading and releasing therapeutics of this ROS‐sensitive hydrogel. Changes in the morphology showed that loading drugs did not affect the formation of hydrogel (Figure S8, Supporting Information). Fluorescence imaging of the hydrogel in which fluorescein isothiocyanate (FITC) was used as the substitution of IPI549 and Cy5.5‐labeled IgG as the substitution of aPDL1^[^ ^37^, ^38^ ^]^ also demonstrated that therapeutics could be successfully encapsulated into gels and displayed uniform distribution (Figure 3G). To observe the drug‐releasing pattern within hydrogels, IPI549 and Cy5.5‐aPDL1 were loaded into the hydrogels. As expected, IPI549 and aPDL1 displayed the accelerated release in H_2_O_2_ solution compared to that in PBS. Notably, most of IPI549 released from the hydrogel within 24 h, whereas aPDL1 had a sustained release rate, with ≈75% released within 4 days (Figure 3H,I). The distinctly sustained release kinetics of IPI549 and aPDL1 was further confirmed in vivo by using a fluorescence imaging system on CT26 tumor‐bearing BALB/c mice. 7 days after subcutaneous injection of scaffold with payloads including indocyanine green (fluorescent surrogated for IPI549) or Cy5.5‐aPDL1, the fluorescence signals were still detected in gel‐scaffold based group, but undetected in the free drug group (Figure 3J), and importantly the signal corresponding to aPDL1 decreased more slowly than that to IPI549 (Figure 3K). The sequential release dynamics of IPI549 and aPDL1 could be utilized to maximize the synergistic anticancer efficacy.

### 2.4 aPDL1&IPI549@Gel for Inhibiting Progression of Residual Tumors after Inadequate Ablation

**Figure 4:**
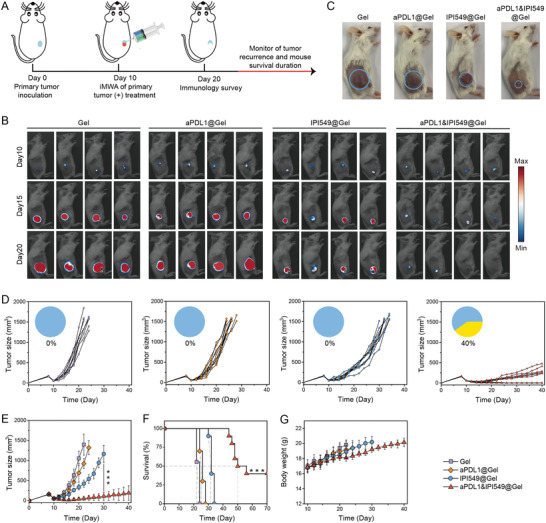
aPDL1&IPI549@Gel inhibiting residual tumor progression post iMWA. A) Schematic illustration of different treatment plans in an inadequate microwave ablation (iMWA) mouse tumor model. B) In vivo bioluminescence images of mice in different groups including Gel, aPDL1@Gel, IPI549@Gel, and aPDL1& IPI549@Gel every 5 days from days 10 to 20. C) Representative mouse photographs at day 14 post different treatments (treatments started at day 10). Blue circles indicate the residual tumors. D) Individual and E) average residual tumor growth kinetics in four groups. Pie charts show percent of complete responses (yellow), and complete response was defined as mouse had no visible or palpable tumor during successive measurements. Data are presented as means ± SD (*n* = 9–10). Statistical significance was calculated by one‐way ANOVA with a Tukey post‐hoc test. ***p < 0.001. F) Kaplan–Meier survival curves of mice as indicated. The dotted line represents median survival time. Statistical significance was calculated via the Log‐Rank test, ***p < 0.001. G) Average body weights of mice in various therapeutics. Data are presented as means ± SD (*n* = 9–10). aPDL1, anti‐programmed death‐ligand 1 blocking antibody.

To validate whether the combination of PI3K*γ* inhibition and PD‐L1 blockade could reduce outgrowth of the residual tumors after inadequate ablation, single doses of this in situ formed gels containing aPDL1 and IPI549 (aPDL1, 50 µg per mouse; IPI549, 25 µg per mouse; gel, 200 µL per mouse) were injected separately at the peritumoral sites (**Figure** **4**A).^[^ ^38^, ^39^ ^]^ Tumor growth of mice was monitored by capturing bioluminescence signals from CT26 cells (Figure 4B and Figure S9, Supporting Information). We observed that the Gel group slowed the growth of residual tumors to some extent compared to PBS group, but had negligible treatment efficacy (Figure S9, Supporting Information). aPDL1@Gel therapy exhibited little effect on suppressing tumor growth (Figure 4C,D), demonstrating that the residual tumor after iMWA was resistant to ICB therapy. Although IPI549@Gel‐treated mice had a modest delay of tumor growth, all of them died within 35 days (Figure 4D). Of note, mice receiving aPDL1&IPI549@Gel treatment showed significant delay of tumor growth (Figure 4E), the median survival was 50 days compared with 22.2 days of Gel group, with complete responses and continuous survival to date in 40% (Figure 4F). Furthermore, the average tumor volume of aPDL1&IPI549@Gel group on day 26 was 16.2‐fold smaller than that of aPDL1&IPI549 group, which should be attributed to the sustained release of aPDL1 and IPI549 from the ROS‐responsive scaffold (Figure S9, Supporting Information). In addition, the mice had no abnormal body‐weight changes after treated with the combined therapeutic strategy (Figure 4G and Figure S9, Supporting Information).

**Figure 5:**
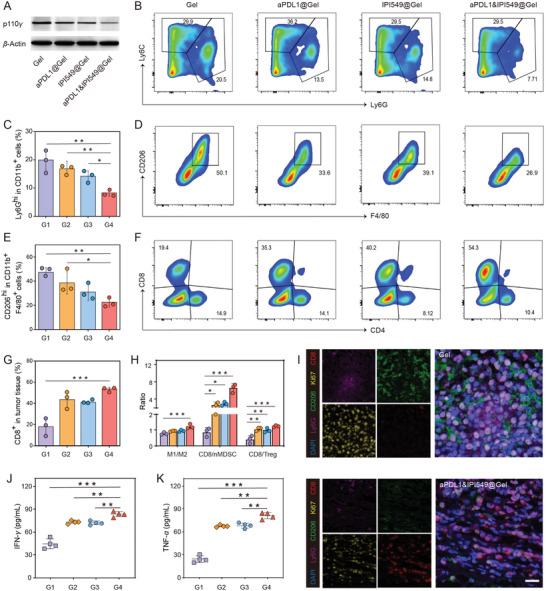
The robust antitumor immune responses triggered by aPDL1&IPI549@Gel. A) Western blot of p110*γ* in residual tumors collected from mice in different groups. B) Representative flow cytometric analysis and C) quantification of nMDSCs (CD11b^+^Ly6G^hi^) in CD45^+^ cells. Data are presented as means ± SD (*n* = 3). D) Representative flow cytometric analysis and E) quantification of TAMs‐M2 (CD206^hi^) in CD11b^+^F4/80^+^ cell population. Data are presented as means ± SD (*n* = 3). F) Representative flow cytometric analysis and G) quantification of CD8^+^ T cells in CD45^+^CD3^+^ cells. Data are presented as means ± SD (*n* = 3). H) Quantification by flow cytometry of M1/M2, CD8/nMDSC, and CD8/Treg ratios. Data are presented as means ± SD (*n* = 3). I) Representative immunofluorescence images of residual tumors displaying CD8^+^ T cell, Ki67, CD206, and Ly6G infiltration. Scale bar: 20 µm. J) Cytokine levels in sera including interferon‐*γ* (IFN‐*γ*) and K) tumor necrosis factor‐*α* (TNF‐*α*) from mice after different treatments. Data are presented as means ± SD (*n* = 4). G1, Gel; G2, aPDL1@Gel; G3, IPI549@Gel; G4, aPDL1&IPI549@Gel. Statistical significance was calculated by one‐way ANOVA with a Tukey post‐hoc test. *p < 0.05, **p < 0.01 and ***p < 0.001. aPDL1, anti‐programmed death‐ligand 1 blocking antibody; nMDSCs, neutrophilic myeloid derived suppressor cells; TAMs, tumor‐associated macrophages; M1, M1‐like macrophage; M2, M2‐like macrophage; Treg, regulatory T cell.

To further reveal the underlying mechanisms of antitumor responses induced by this localized aPDL1&IPI549@Gel immunotherapy, residual tumors were collected on day 10 after injection of various formulations. Western blot of representative tumors revealed that the IPI549‐loaded gels did inhibit the levels of PI3K*γ* (**Figure** **5**A). The expression of PD‐L1 in residual tumor of aPDL1&IPI549@Gel group showed the most significant suppression in comparison with other control groups (Figure S10, Supporting Information), most likely due to a synergistic effect of PI3K*γ* inhibition and ICB therapy. Accordingly, we evaluated changes in the intra‐tumoral leukocyte phenotypes when PI3K*γ* was systemically inhibited by IPI549. All the immunosuppressive cellular components including TAMs‐M2 (CD11b^+^F4/80^+^CD206^hi^), monocytic myeloid derived suppressor cells (mMDSCs; CD11b^+^Ly6C^hi^), neutrophilic myeloid derived suppressor cells (nMDSCs; CD11b^+^Ly6G^hi^) and regulatory T cells (Tregs) were studied by flow cytometry. Although mMDSCs and Tregs were not significantly affected (Figures S11 and S12, Supporting Information), we observed a significant reduction of nMDSCs and TAMs‐M2 in the aPDL1&IPI549@Gel‐treated mice compared to that in the control ones (Figure 5B–E). Additionally, the locally combined immunotherapy could effectively enhance the frequency of tumor‐infiltrating CD8^+^ T cells (CD3^+^CD4^−^CD8^+^) but not CD4^+^ T cells (CD3^+^CD4^+^CD8^−^) or TAMs‐M1 within the residual tumors (Figure 5F,G and Figure S13, Supporting Information). Notably, the ratios of M1/M2, CD8/Treg and CD8/nMDSC, which could be recognized as indicators of anticancer immune balance, were detected to be the highest in aPDL1&IPI549@Gel group, consistent with the obtained strongest antitumor effects in this group (Figure 5H). These results were further confirmed by mIHC analysis, which indicated that the residual tumor in Gel group had high proportions of myeloid cells including nMDSCs and TAMs‐M2, as well as a limited CTL infiltration. In contrast, aPDL1&IPI549@Gel resulted in an effective increase in the number of CD8^+^ T cells, but a decreased proportion of myeloid cells (Figure 5I and Figure S14, Supporting Information). Also, the Ki67 expression was significantly reduced in the treated group, demonstrating that the combination therapy effectively impeded tumor proliferation (Figure 5I). Furthermore, we found an increase of interferon‐*γ* (IFN‐*γ*) and tumor necrosis factor‐*α* (TNF‐*α*) in sera of mice treated with aPDL1&IPI549@Gel (Figure 5J,K), which once again verified the robust immune responses stimulated by such an immunotherapy strategy.

In addition, the assessment of the toxic effects of combination therapies should be always given the serious concern. As thus, serum biochemistry assay and histology analysis of major organs were conducted at day 10 and day 20 after receiving hydrogels loaded with IPI549 and aPDL1 (Figures S15,S16, Supporting Information). As a matter of fact, all the measured indexes of combined therapy group maintained in the same ranges as those of the healthy controls, indicating that the proposed aPDL1&IPI549@Gel therapy induced no appreciable in vivo systemic toxicity at the chosen experimental dose.

### 2.5 aPDL1&IPI549@Gel Induces Systemic Immune Responses

**Figure 6:**
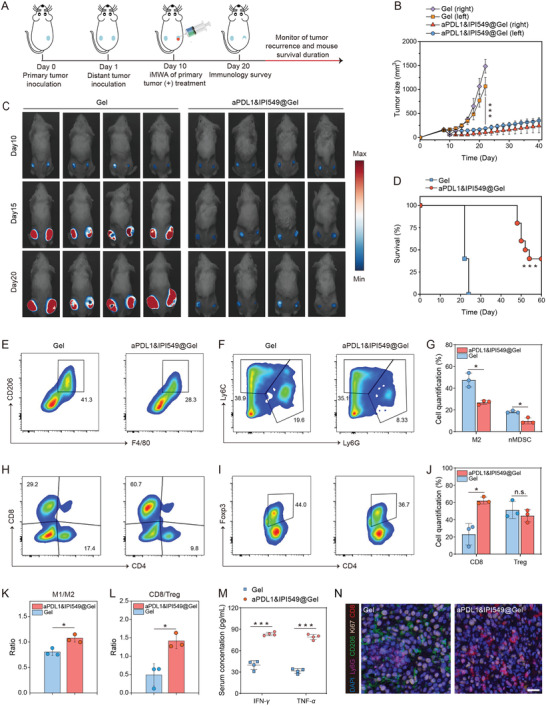
aPDL1&IPI549@Gel stimulating the systemic immune responses after iMWA. A) Schematic illustration of combination therapy to inhibit distant tumor growth in a mouse model of inadequate microwave ablation (iMWA). B) The left and right tumor growth kinetics. Data are presented as means ± SD (*n* = 10). C) In vivo bioluminescence images of mice in Gel and aPDL1&IPI549@Gel group every 5 days from days 10 to 20. D) Kaplan–Meier survival curves of mice as shown. Statistical significance was calculated via the Log‐Rank test, ***p < 0.001. E) Representative flow cytometric analysis of TAMs‐M2 (CD206^hi^) gating on CD11b^+^F4/80^+^ cells, F) nMDSCs (CD11b^+^Ly6G^hi^) gating on CD45^+^ cells and G) their corresponding flow cytometric quantification. Data are presented as means ± SD (*n* = 3). H) Representative flow cytometric analysis of CD8^+^ T cells gating on CD45^+^CD3^+^ cells, I) CD4^+^Foxp3^+^ T cells gating on CD3^+^ cells, and J) their corresponding flow cytometric quantification. Data are presented as means ± SD (n  =  3). K) Quantification by flow cytometry of ratios of M1/M2 and L) CD8/Treg. Data are presented as means ± SD (*n* = 3). M) Cytokine levels in sera including interferon‐*γ* (IFN‐*γ*) and tumor necrosis factor‐*α* (TNF‐*α*) from mice isolated at 10 days after Gel and aPDL1&IPI549@Gel treatment. Data are presented as means ± SD (*n* = 4). N) Representative immunofluorescence images of distant tumors displaying CD8^+^ T cell, Ki67, CD206, and Ly6G expression 10 days after Gel and aPDL1&IPI549@Gel injection. Scale bar: 20 µm. Statistical significance was calculated by Student's *t* test. n.s, not significant, *p < 0.05, ***p < 0.001. aPDL1, anti‐programmed death‐ligand 1 blocking antibody; nMDSCs, neutrophilic myeloid derived suppressor cells; M1, M1‐like macrophage; M2, M2‐like macrophage; Treg, regulatory T cell.

We further assessed whether this kind of local treatment could inhibit distant tumor progression by activating systemic anticancer immunity. CT26 cancer cells inoculated in the right flank of each mouse were set as the primary tumor. 1 day after the primary tumor was transplanted, a second tumor was inoculated in the left flank of the same mouse to mimic metastatic tumor. On day 10, the primary tumors were treated with iMWA, and subsequently Gel (200 µL per mouse) or aPDL1&IPI549@Gel (aPDL1, 50 µg per mouse; IPI549, 25 µg per mouse) were injected at the peri‐tumoral sites (**Figure** **6**A). We observed that both sides of tumors in Gel group grew at an uncontrollable rate, and the right tumors receiving iMWA grew more rapidly than the left ones without any intervention. On the contrary, the combination therapy to some extent inhibited progression of the primary tumors and significantly slowed down the outgrowth of mimic distant tumors (Figure 6B,C and Figures S16,S17, Supporting Information). Besides, weights of mice were closely monitored as displayed in Figure S17, Supporting Information. Congruently, the overall survival of mice after aPDL1&IPI549@Gel treatment was substantially prolonged when compared to Gel control, proving that such a combined immunotherapy strategy is highly effective for cancer metastases treatment (Figure 6D).

Congruent with the systemic anticancer responses triggered by anti‐PI3K*γ* + anti‐PD‐L1 therapy, we observed a substantial decrease of myeloid suppressor cells including TAMs‐M2 (but not TAMs‐M1) and MDSCs (refer to nMDSCs, not mMDSCs) in the distant tumors 10 days after treatments, comparable to control tumors (Figure 6E‐G and Figure S18, Supporting Information). Concomitantly, the number of intratumoral CD8^+^ T cells (but not CD4^+^ T cells) was increased undergoing combination therapy (Figure 6H and Figure S18, Supporting Information). Also, there was a slight but statistically insignificant decrease in the frequency of Tregs in the distant tumors treated with aPDL1&IPI549@Gel (Figure 6I,J). Thus, as mentioned above, the anticancer immune balance indicators such as M1/M2, CD8/Treg, and CD8/nMDSC ratios were all obviously improved in the IPI549/aPDL1 combination therapy group (Figure 6K,L and Figure S19, Supporting Information). Consistent with the above results, cytokines including IFN‐*γ* and TNF‐*α* which played important roles in cellular antitumor immunity increased significantly (Figure 6M). In support, mIHC confirmed that in the aPDL1&IPI549@Gel group, relatively lower proportions of TAMs‐M2 and nMDSCs were observed in the distant tumors while in parallel with a large number of CD8^+^ T cells infiltration, as compared to the untreated control on day 10 (Figure 6N). Together, these results demonstrated that an effective immune response was induced by aPDL1&IPI549@Gel treatment.

### 2.6 aPDL1&IPI549@Gel Induces Durable Immune‐Memory Effects

**Figure 7:**
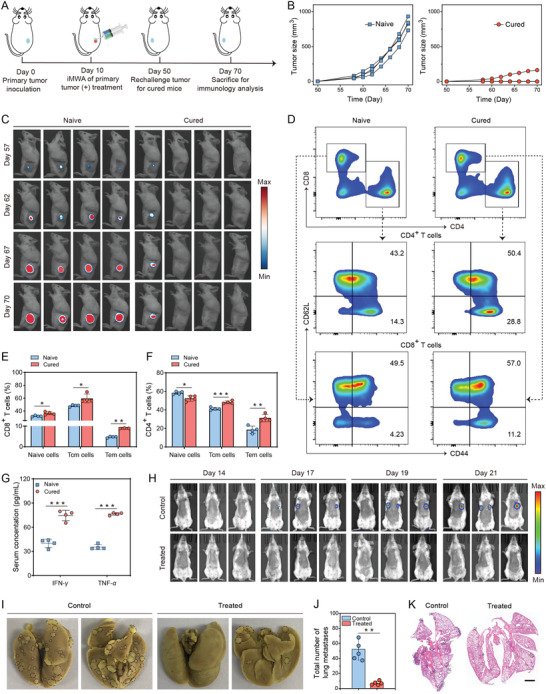
aPDL1&IPI549@Gel inducing T cell memory against tumor‐rechallenge and inhibiting the whole‐body spreading metastasis. A) Schematic illustration for evaluating the immune memory effects of the cured mice receiving aPDL1&IPI549@Gel treatment. B) Individual tumor growth kinetics of the naive and cured mice. (*n* = 4). C) In vivo bioluminescence images of the naive and cured mice. D) Representative flow cytometric analysis of central memory T (Tcm) cells and effector memory T (Tem) cells gating on CD3^+^ cells. Data are presented as means ± SD (*n* = 4). E,F) Flow cytometric quantification of naive T cells, Tcm cells, and Tem cells in the spleen. Data are presented as means ± SD (*n* = 4). G) Interferon‐*γ* (IFN‐*γ*) and tumor necrosis factor‐*α* (TNF‐*α*) levels in serum obtained from mice of the naive group and cured group. H) In vivo bioluminescence images to track the outgrowth of fLuc‐CT26 tumor cells after different treatments. I) Representative photographs showing the metastatic tumor nodules in the excised lung tissues. J) Quantification of lung metastasis. Data are presented as means ± SD (*n* = 5). K) Representative hematoxylin and eosin (H&E) staining analysis of the lung tissues. Statistical significance was calculated by Student's *t* test. *p < 0.05, **p < 0.01 and ***p < 0.001. aPDL1, anti‐programmed death‐ligand 1 blocking antibody.

Immune‐memory response is a hallmark of adaptive immunity through which the immune system can remember pathogens that have invaded organisms before and confer durable immunity, and it plays a crucial role in protecting organisms from a second pathogen attack.^[^ ^40^ ^]^ To assess whether this combined tumor therapy could induce long‐term adaptive immunity, we rechallenged the cured mice after aPDL1&IPI549@Gel treatment in the unilateral tumor model part with fLuc‐CT26 cells in the opposite flank on day 50, with sex‐ and age‐matched naive mice injected equal number of tumor cells as controls (**Figure** **7**A). As expected, all the naive mice eventually developed large tumors (Figure 7B), in contrast, the outgrowth of re‐inoculated tumors in combined treatment group was significantly impeded. Notably, three out of four mice after combined treatment were completely resistant to the rechallenge (Figure 7C and Figure S20, Supporting Information). These results revealed that the long‐lasting immune‐memory effects were generated by the proposed aPDL1&IPI549@Gel combined immunotherapy.

To further understand the underlying immunological mechanism by which aPDL1&IPI549@Gel induced such long‐term memory responses, spleens and serum samples of mice were collected 20 days after the rechallenge. It is well known that based on the proliferative capacity, effector function, and migration potential, antigen‐specific memory T cells are classified into central memory T (Tcm, CD3^+^CD8^+^CD62L^+^CD44^+^) cells and effector memory T (Tem, CD3^+^CD8^+^CD62L^−^CD44^+^) cells subsets.^[^ ^41^ ^]^ Tcm exhibits a strong proliferative potential and can provide protections after several immunoediting processes including expansion, differentiation, and trafficking.^[^ ^42^ ^]^ Compared with Tcm,^[^ ^42^ ^]^ Tem can respond quickly and provide immediate protections when exposed to the secondary encountered tumor antigen by producing cytokines like IFN‐*γ* and TNF‐*α*.^[^ ^43^ ^]^ Intriguingly, we found that the proportions of naive, Tcm, and especially Tem in CD8^+^ T cells were significantly elevated in the long‐term survival mice compared with the naive mice (Figure 7D,E). Similarly, for the CD4^+^ T cell population, although the percentages of naive T cells of the cured group were a little lower, numbers of both Tem and Tcm were much higher than those of the naive group (Figure 7D,F). Furthermore, production of serum cytokines including IFN‐*γ* and TNF‐*α* was significantly augmented with combination therapy (Figure 7G). Taken together, these findings provided critical evidence that a durable immune‐memory effect was generated by local delivery of aPDL1&IPI549@Gel and that was highly effective to prevent tumor recurrence.

### 2.7 aPDL1&IPI549@Gel Inhibits the Whole‐Body Spreading Metastasis

Encouraged by the excellent therapeutic performance of aPDL1&IPI549@Gel treatment in inhibiting progression of the primary and distant tumors, as well as resisting tumor rechallenge, we further evaluated the efficacy of our strategy with an aggressive whole‐body spreading tumor model. In this study, unilateral tumor‐bearing mice were intravenously inoculated with fLuc‐CT26 tumor cells 1 day before the ablated tumors were eliminated by aPDL1&IPI549@Gel immunotherapy deriving from IPI549‐mediated immunosuppressive subversion and anti‐PD‐L1 blockade. As shown in Figure 7H, bioluminescence signals of cancer metastasis were detected in the control mice on the 17th day after intravenous injection of tumor cells. With the feeding duration prolonging, augmented fluorescence signals were detected in all the mice of this group on the 21st day. In contrast, the mice treated with aPDL1&IPI549@Gel only exhibited stellate bioluminescence signals, indicating that the spreading metastasis could be substantially suppressed (Figure 7H).

Accordingly, gross appearance of the excised lung tissues directly revealed that compared with the untreated controls, mice receiving aPDL1&IPI549@Gel combined treatment exerted a noticeable effect on preventing lung metastasis (Figure 7I). The quantitative results verified that the combined immunotherapy strategy substantially reduced the number of lung nodules compared with the control group, that is, 7.0 ± 2.7 lung nodules versus 50.2 ± 12.8 lung nodules (Figure 7J). These results were then further validated with pathological analysis by using H&E staining which clearly showed a great number of metastatic tumor nodules occupied in lungs in the controls but only very few lung nodules in the aPDL1&IPI549@Gel treatment group (Figure 7K). These data further claimed that this Gel‐based combined PI3K*γ* and PD‐L1 inhibition immunotherapy strategy was beneficial for establishing powerful anticancer immunity after iMWA to the whole‐body spreading metastatic CT26 tumors, represented by lung metastasis.

### 2.8 aPDL1&IPI549@Gel Inhibits Tumor Recurrence in 4T1 Cancer Models

After demonstrating the robust and effective antitumor immune response of the ROS‐responsive scaffold in CT26 tumor type, we wondered whether such a localized immunotherapy approach could be effective for other types of tumors. In our work, we established 4T1 cancer model in BALB/C mice, and the treatment plan was the same as the aforementioned protocol. Encouragingly, we found that aPDL1&IPI549@Gel treatment could inhibit significantly tumor recurrence post iMWA (Figure S21, Supporting Information). These results indicate that our localized immunotherapy method in the CT26 cancer models can be extended to other tumor types.

## 3 Conclusions

Growing evidence has highlighted that the combination of loco‐regional PTA and immunotherapy especially for ICB is a promising approach for cancer treatment.^[^ ^11^ ^]^ Thus, it is instructive to study how to integrate them reasonably and scientifically. Herein, with a preclinical CT26 colon adenocarcinoma murine model, we discovered that iMWA of a target tumor accelerated the tumor progression mainly owing to the TAMCs‐mediated immune suppression. Relatedly, the gene signature of injured tissue post‐ablation was highly correlated with ICB resistance in clinic.^[^ ^30^ ^]^ Given the major role of PI3K*γ* in myeloid cells, these findings strongly support the speculation that combining PI3K*γ* inhibition with ICB therapy should likely yield additional antitumor activity after insufficient thermal ablation. Therefore, we rationally designed a bioresponsive scaffold‐based immunotherapy strategy capable of actively subverting TAMCs‐induced immunosuppression via a selective pharmacological PI3K*γ* inhibitor (IPI549) and subsequently promoting ICB‐mediated immune response, ultimately revolutionizing the PTA‐based antitumor therapies. This in situ scaffold system offers a convenient and effective approach that can be seamlessly integrated with clinical image‐guided PTA procedure without the need for additional operation. Our results showed that growth of CT26 colon tumors after suboptimal, partial MWA treatment could be significantly inhibited and even entirely eradicated with the addition aPDL1&IPI549@Gel therapy, which also offered an obvious survival benefit. Furthermore, treatment with this combination therapy affected both untreated distant and spreading tumors, and importantly, it induced a long‐lasting adaptive antitumor immunity for tumor‐free survivors resistant to tumor re‐implantation. Thus, it can be regarded as an appealing therapeutic approach holding high promise for maximizing the clinical outcome of widely applied PTA therapies.

A unique advantage of the present study is that we propose, establish and demonstrate a precision medicine strategy in which the design of immunotherapeutic combination is modified based on the tumor immune landscape to overcome these resistance mechanisms. Actually, although one aims for full ablation, it cannot be always achieved. The target tumor may experience inadequate ablation because it is difficult to achieve complete tumor destruction due to larger tumor size or proximity to the gastrointestinal organs and large vessels.^[^ ^44^, ^45^ ^]^ Considering the international guidelines‐based wide application of PTA in clinical practice, clarifying the biological effects of thermal ablation on residual tumors seems to be extremely important. Surgery‐induced wound‐healing response has been proved to be causative of the tumor progression and metastasis.^[^ ^46^, ^47^ ^]^ Consistently, we demonstrated that iMWA could promote proliferation of residual CT26 cancer cells. Although various tumorigenesis factors such as interleukin‐6 (IL‐6), c‐met, or hepatocyte growth factor (HGF)/Hypoxia‐inducible factor‐1*α* (HIF‐1*α*)/VEGF*α*‐dependent pathways have been shown to be associated with the promoted tumor growth of subtotal thermal ablation, ours is the first to more thoroughly examine the differences of residual tumors before or after iMWA in RNA signatures and in‐depth immunophenotyping. And on the basis of these unbiased findings, we further established this gel‐based TAMCs targeting plus ICB therapy for ablative colon adenocarcinoma. Going forward, our proposed periablative setting should represent a high‐leverage context in which to permissively administer PTA‐based anticancer immunotherapy.

Another advantage of this localized immunotherapy method is to achieve systemic anticancer immunity after the local implantation of immunotherapeutic scaffold. These excellent results may be attributed to two main reasons. On the one hand, the in situ gelation involved in this strategy enables local retention and controlled sequential release of therapeutics (IPI549 and aPDL1). IPI549 has a smaller molecular weight compared to aPDL1, which results in its faster release from the hydrogel. IPI549 release first could modulate the myeloid cell‐induced immunosuppression. By doing so, sensitivity to PD‐L1 blockade could be effectively restored in the myeloid cell‐rich tumors, which should be vial for maximizing the synergistic anticancer efficacy. Although ICB seemed to be tolerated by patients, the combination therapies might enhance the risk of medication‐related side effects. In this study, the adopted PVA is considered to be highly biocompatible and excreted from the body through biliary tract excretion.^[^ ^48^ ^]^ And no obvious systemic toxicity was observed in tumor‐bearing mice treated with these drug‐loaded scaffolds. Indeed, the convergence of PTA, cancer immunotherapy, nanotechnology, and drug delivery system nowadays is opportune,^[^ ^20^, ^24^, ^25^ ^]^ as each of the fields has independently matured to the point that it could now be utilized to complement the others. On the other hand, as highlighted herein, targeting a key pathway in TAMCs may help to extract maximum efficiencies from the combined treatment. Actually, numerous chemokines such as CC chemokine ligand 2 (CCL2) and CC chemokine ligand 4 (CCL4), as well as IL‐1b and IL‐6, and VEGF*α* and cytokines colony‐stimulating factor (CSF)‐1, have been proved to promote the myeloid cell recruitment to tumors in preclinical mouse models of breast, lung, and pancreatic cancer.^[^ ^49^ ^]^ Of note, PI3K*γ* functions downstream of the above diverse chemoattractant receptors.^[^ ^19^, ^49^ ^]^ As thus, compared with only selective chemoattractant‐blockade, blocking PI3K*γ* signaling pathway that is common to various chemoattractant receptors could enable a more targeted administration of the myeloid cell trafficking.

Finally, the therapeutic approach we offered should likely be a common framework for powerfully complementing insufficient loco‐regional treatment that is not confined to PTA (e.g., MWA, RFA, and LA), but surgery and radiotherapy. Data from many preclinical and clinical studies have shown that surgical wounding‐triggered inflammation is anticancer in nature but also immunosuppressive as a result of the recruitment of immune suppressive cells especially for myeloid cells.^[^ ^46^, ^47^ ^]^ Also, a preclinical study using colon cancer model confirms radiotherapy can dramatically enhance infiltration of TAMCs into tumor sites that strongly suppresses radiotherapy‐elicited immune responses and is also recognized as a crucial resistance mechanism to radiotherapy.^[^ ^50^ ^]^ We presented an immunotherapeutic strategy that could modulate the suppressive leukocyte phenotype within the postablative microenvironment toward a more immunostimulatory one by selectively targeting the myeloid PI3K*γ* isoform with a pharmacologic inhibitor. Excitingly, it brought distinct synergistic effects when blended with PD‐L1 blockade immunotherapy, which not only prevented local tumor progression but also led to the inhibition of existing metastases. With deductive intention, such a bioengineering‐based therapeutic strategy has the potential to be set as a standard‐of‐care paradigm for revolutionizing the treatment of TAMCs‐rich residual tumors after other conventional therapies.

In conclusion, our data presented here offer strong evidence for the complex inflammatory reactions triggered by iMWA, which can impose an aggressive phenotype on the nearby tumor residues mainly attributed to the high infiltration of suppressive TAMCs. These unbiased findings have provided a strong rationale to further consider assessing PI3K*γ* inhibition in combination with ICB for postablative treatment on the basis of a precision‐medicine‐type evaluation of the tumor immune landscape. More specifically, we have established and engineered a viable hydrogel‐immunotherapy approach for cancer postablative treatment by administration of ROS‐responsive scaffold containing aPDL1&IPI549 at the tumor‐ablated sites. By employing five tumor models, we have proved that aPDL1&IPI549@Gel strategy can not only efficiently suppress the primary tumor growth treated locally with thermal ablation but also substantially prevent the distant and lung metastasis. Owing to the stimulation in T memory cells, our combined immunotherapy strategy can protect against tumor rechallenge after the initial tumor elimination. As a widely accepted supply of PTA in clinical practice, successful translation of this strategy would greatly impact how, when, and where anticancer immunotherapy is delivered in interventional oncology.

## Conflict of Interest

The authors declare no conflict of interest.

## Supporting information

Supporting Information Materials

## Data Availability

The data that support the findings of this study are available from the corresponding author upon reasonable request.
