# Supplementary material for: Engineering ROS‐Responsive Bioscaffolds for Disrupting Myeloid Cell‐Driven Immunosuppressive Niche to Enhance PD‐L1 Blockade‐Based Postablative Immunotherapy
Source: Adv Sci (Weinh). 2022 Feb 13;9(11):2104619. doi: 10.1002/advs.202104619 (PMC9008797; doi:10.1002/advs.202104619)
Supplement: Supplementary file 1 — Supporting Information Materials [file ADVS-9-2104619-s001.pdf]

ADVANCED SCIENCE
Open Access

Supporting Information

for *Adv. Sci.,* DOI: 10.1002/advs.202104619

Engineering ROS-responsive bioscaffolds for disrupting myeloid cell-driven immunosuppressive niche to enhance PD-L1 blockade-based postablative immunotherapy

Shaoyue Li, Chunyan Zhu, Xianli Zhou, Liang Chen, Xiaowan Bo, Yuting Shen, Xin Guan, Xiaoxia Han, Dandan Shan, Liping Sun, Yu Chen, Huixiong Xu*, Wenwen Yue**

Copyright WILEY-VCH Verlag GmbH & Co. KGaA, 69469 Weinheim, Germany, 2021.

Engineering ROS-responsive bioscaffolds for disrupting myeloid cell-driven immunosuppressive niche to enhance PD-L1 blockade-based postablative immunotherapy

Shaoyue Li, Chunyan Zhu, Xianli Zhou, Liang Chen, Xiaowan Bo, Yuting Shen, Xin Guan, Xiaoxia Han, Dandan Shan, Liping Sun, Yu Chen, Huixiong Xu*, Wenwen Yue**

S. Li, C. Zhu, X. Bo, Y. Shen, X. Guan, X. Han, D. Shan, Prof. L. Sun, Prof. Y. Chen, Prof. H. Xu, Dr. W.Yue

Department of Medical Ultrasound, Shanghai Tenth People's Hospital, Ultrasound Research and Education Institute, School of Medicine, Tongji University, Shanghai Engineering Research Center of Ultrasound Diagnosis and Treatment, National Clinical Research Center of Interventional Medicine, Shanghai, 200072, P. R. China

E-mail: yuewen0902@tongji.edu.cn; xuhuixiong2022@126.com; chenyuedu@shu.edu.cn

Prof. H. Xu

Department of Ultrasound, Zhongshan Hospital, Fudan University, Shanghai, 200032, P. R. China

S. Li, Prof. X. Zhou

Department of In-patient Ultrasound, The Second Affiliated Hospital, Harbin Medical University, Harbin, 150001, P. R. China

Dr. L. Chen

Department of Gastroenterology, Shanghai Tenth People's Hospital, Tongji University School of Medicine, Shanghai, 200072, P. R. China

Prof. Y. Chen

Materdicine Lab, School of Life Sciences, Shanghai University, Shanghai, 200444, P. R. China

S. Li., C. Zhu. and X. Zhou. contributed equally to this work.

A: Experimental section

Study design. Percutaneous thermal ablation (PTA) has been well established as one of the most widely utilized therapeutic interventions for at least 12 distinct cancer types and showed significant magnitude of benefit supported by several international clinical guidelines. However, inadequate ablation still remains a therapeutic dilemma for any loco-regional treatment. Accordingly, the objective of this study was to investigate the potential principles responsible for such pro-oncogenic effects derived from inadequate ablation and further provide a precise combined immunotherapy strategy to maximize the clinical effects of PTA treatment. We first utilized a preclinical colon adenocarcinoma murine model to confirm that inadequate ablation indeed stimulated outgrowth of residual tumor and we discovered the mechanism that tumors were exposed to myeloid cell-mediated tumor immune microenvironment (TIME) after inadequate microwave ablation (iMWA). Then, a myeloid cell-targeted combined strategy was designed based on a ROS-responsive scaffold and its anticancer efficacy was evaluated through multiple types of mouse models. Mice from different treatment groups were measured and imaged to detect tumor progression and metastasis, and were also rechallenged with tumor cells to evaluate immune memory effects. Animals were euthanized by carbon dioxide for signs of cachexia or when the tumor volume reached 1500 mm^3.

Materials. All chemicals in our study were obtained from Sigma-Aldrich unless otherwise specified. IPI549 was purchased from MedChemExpress (Catalog No. HY-100716). Anti-PD-L1 was supplied by Bioxcell (α-PD-L1, Clone: 10 F.9G2, Catalog No. BE0101)

Cell lines and mice. All mouse experiments for the whole study were performed according to protocols in accordance with the policies of the National Ministry of Health and approved by the Laboratory Animal Center of Shanghai Tenth People's Hospital. CT26 murine colorectal cancer-cell line and 4T1 murine breast cancer-cell line were purchased from Shanghai Institutes for Biological Sciences. fLuc-CT26 cancer-cell line was purchased from Shanghai Zhong Qiao Xin Zhou Biotechnology Co. Ltd. All cell lines used in this work were quarterly authenticated by unique STR profile and results indicated no cross-contamination. Cells were maintained in the recommended medium and condition. Female BALB/c mice aged 6-8 weeks were purchased from Ling Chang Biological Technology Co. Ltd.

Formation of TSPBA. N,N,N′,N′-tetramethyl-1,3-propanediamine (0.2 g, 1.5 mmol) and 4-

(bromomethyl) phenylboronic acid (1 g, 4.6 mmol) were added in dimethylformamide (DMF) (40 mL) and stirred using a water bath with magnetic stirring for 24 h at 60°C. Next, the clarified solution was poured into tetrahydrofuran (THF) (100 mL). The obtained white sediment was washed with THF (20 mL) for three times. After drying under the vacuum and low temperature conditions overnight, pure TSPBA (0.6 g, yield 70%) was obtained. TSPBA was characterized by ^{1}H-NMR spectroscopy (300 MHz; solvent: d-DMSO, D_2O).

Synthesis of PVA-TSPBA hydrogels. PVA (72 kDa; 98% hydrolyzed; 1 g) and deionized water (20 mL) were mixed and stirred together in a water bath with magnetic stirring. The temperature was raised slowly up to 95°C in order to achieve a clear solution. TSPBA (5 weight % (wt %) in H_2O, 2 mL)) and PVA (5 wt % in H_2O, 2 mL) were mixed to fabricate a tough hydrogel. The gels were divided into several copies for *in vitro* experiments. For the fabrication of aPDL1- and IPI549-loaded gel, aPDL1 (50 μg per sample) and IPI549 (25 μg dissolved at 5% 1-methyl-2-pyrrolidinone in polyethylene glycol 400 per sample) were dissolved in PVA aqueous solution. For *in vivo* experiments, PVA (with or without drugs) and TSPBA solutions were first kept at room temperature for 30 minutes, then double tube syringe with 0.45 mm-diameter needle was used to form gels by injecting PVA and TSPBA solutions in a volume ratio of 1:1.

Characterization of PVA-TSPBA hydrogels. After hydrogels were frozen at -80 °C overnight, the microstructure of gels was performed by cryo-scanning electron microscopy (Cryo-SEM) (SU8010, Japan). The dynamic rheological behavior of PVA aqueous solution before and after gelation was measured at 25°C using a Thermo HAAKE MARS 60 stress-controlled rheometer with 20 mm parallel plates.

In vitro and in vivo hydrogels degradation. For *in vitro* experiment, gels were put in PBS with or without H_2O_2 (1 mM, 0.5 mM, 0.25 mM) and pictured on day 0, 3 and 7 to observe their morphology changes. For *in vivo* experiment, gels (200 μL per sample) were injected subcutaneously into BALB/c mice. Pictures were taken and mice were sacrificed on day 0, 3, 7 and 14 for hematoxylin and eosin (H&E) staining of the surrounding skin in order to record degradation behavior and tissue biocompatibility.

In vitro and in vivo release of aPDL1 and IPI549 from hydrogels. The *in vitro* drug release studies from ROS-responsive hydrogels were performed in PBS with or without H_2O_2 (1 mM) at room temperature. The released IPI549 and Cy5.5-aPDL1 were analyzed using ultraviolet spectrum and SpectraMax iD5, respectively. To evaluate the *in vivo* release of

aPDL1, free Cy5.5-aPDL1 or Cy5.5-aPDL1@Gel was injected subcutaneously into CT26 tumor-bearing BALB/c mice. On day 0, 3 and 7, fluorescence imaging of Cy5.5-aPDL1 release was monitored by the IVIS imaging system. Likewise, for the assessment of IPI549 release, representative fluorescence imaging of a drug-loaded gel in which indocyanine green was used as a fluorescent surrogate for IPI549 that was taken *via* the IVIS imaging system.

Tumor models and treatment experiments. 1 × 10^6 of fLuc-CT26 cells or 1 × 10^6 of 4T1 cells were injected intradermally into each BALB/c mouse in the right flank. When the longest diameter of tumor reached about 0.8 cm (ten days after tumor implantation), the inadequate ablation was started by using a microwave generator (ECO-100E, Yigao Microwave Electric Institute, Nanjing, China). After the animal was anesthetized, the MWA antenna was inserted percutaneously into the central position of the tumor on the long axis under sterile conditions. Treatments were controlled for 1.0-1.5 min with the ablation power set at 5 Walt. The whole ablation operations were finished by two operators. The mice were divided randomly into six groups and peritumorally implanted with different formulations, including PBS (200 μL per mouse), Gel (200 μL per mouse), aPDL1@Gel (aPDL1, 50 μg per mouse), IPI549@Gel (IPI549, 25 μg per mouse), aPDL1&IPI549 (aPDL1, 50 μg per mouse; IPI549, 25 μg per mouse) and aPDL1&IPI549@Gel (aPDL1, 50 μg per mouse; IPI549, 25 μg per mouse). Bioluminescence signals from cancer cells were taken *via* the IVIS imaging system to assess the residual tumor progression. The size of the tumors was measured every second day with a caliper, and the volume was calculated using the formula, $(L \times W^2)/2$, where L is the longest diameter of tumor and W is the perpendicular diameter. Mice were weighed and arranged to evaluate survival curves. Mice that no visible tumor could be measured on continuous measurement days were considered as complete regressions. For the rechallenged study, on day 50 since primary tumor implantation, cured mice after aPDL1&IPI549@Gel treatment were rechallenged with 5×10^5 fLuc-CT26 cells tumor cells on their opposite flanks.

For the distant tumor model, one day after 1×10^6 fLuc-CT26 cells suspended in PBS were inoculated into the right flank of mice, a second tumor as the mimic distant tumor (1×10^6 fLuc-CT26 cells) was subcutaneously inoculated into the left flank of each mouse. Ten days later, tumors in the right flank were received iMWA treatment and then tumor-bearing mice were divided randomly into two groups, and Gel or aPDL1&IPI549@Gel was injected peritumorally in the right site, respectively, while no treatment was performed for the left tumor site. The subsequent monitoring of mouse bilateral tumors and survival duration was the same as aforementioned procedures.

To establish lung metastases, 1 × 10^6 of CT26 tumor cells were injected intradermally into the BALB/c mouse in the right flank, and sex- and age- matched healthy mice were chosen as controls. Nine days later, all the mice were inoculated intravenously with fLuc-CT26 cells (1 × 10^5) *via* tail vein infusion. On the following day, when the longest diameter of tumor reached about 0.8 cm, the primary tumor of each mouse was treated with iMWA. The followed immunotherapy strategy of treatment group was the same as above. The bioluminescence imaging was carried out using the IVIS imaging system with 60 seconds exposure time to record the state of lung metastases. At the end of this experiment, lungs were collected and fixed in Bouin's solution for 24 h. Pictures of lung tissues were taken with a digital camera, and then the lungs were studied by pathological analysis.

In vivo bioluminescence and imaging. The progression of tumor was observed using the IVIS Smart Imaging System (Vieworks Co. Ltd. Korean). Ten minutes after intraperitoneal injection of d-luciferin dissolved in PBS (15 mg mL^{-1}) at a dose of 10 μL g^{-1}, mice were anesthetized and photographed by the imaging system for 60 seconds of exposure time. Bioluminescence images were then analyzed using IVIS Living Image software. Regions of interest were quantified as average radiance (photons s^{-1} cm^{-2} sr^{-1}).

RNA-sequencing. Tumors were isolated from the mice 3 days after treated with iMWA and RNA was extracted using HiPure Unviersal RNA Mini Kit (Magen Biotechnology Co. Ltd, China). RNA sequencing was performed using RNA Nano 6000 Assay Kit of the Agilent Bioanalyzer 2100 system (Agilent Technologies, CA, USA). log_2(Count) was calculated for each gene, and the data were mean centred for display in the heatmaps.

Cytokine analysis. The serum was collected from mice after different treatments. The serum levels of interferon-*γ* (IFN-*γ*) (Invitrogen, Catalog No. BMS606INST) and tumor necrosis factor-*α* (TNF-*α*) (Invitrogen, Catalog No. BMS607-3) were measured with enzyme-linked immunosorbent assay (ELISA) kits according to the manufacturer's instructions.

Flow cytometry staining and analysis. To study the immune cells in the experiment, cells isolated from mouse tumors and spleens were homogenized into single cell suspensions (10^6 cells in 100 μL total volume). Then, the harvested cells were pre-incubated (15 min, 4°C) with anti-CD16/32 monoclonal antibody to block nonspecific binding and then stained with 100 times diluted of the following fluorochrome-conjugated antibodies: CD45-eF506 (eBioscience, Catalog No. 69-0451), CD11b-APC-Cy7 (eBioscience, Catalog No. 25-0112), Gr-1-APC (Biolengend, Catalog No. 108412), Ly6G-PE (eBioscience, Catalog No. 12-9668),

Ly6C-PerCP-Cy5.5 (eBioscience, Catalog No. 45-5932), F4/80-BV421 (Biolengend, Catalog No. 123137), CD206-FITC (Biolengend, Catalog No. 141703), CD80-APC (Biolengend, Catalog No. 104714), CD3-PE-Cy7 (eBioscience, Catalog No. 25-0031), CD4-FITC (eBioscience, Catalog No. 11-0041), CD8-PerCP-Cy5.5 (eBioscience, Catalog No. 45-0081) and Foxp3-PE (eBioscience, Catalog No. 12-5773) and then analyzed by flow cytometry (FCM). To analyze the memory T cells, single cell suspensions from spleen were stained with CD3-PE-Cy7 (eBioscience, Catalog No. 25-0031), CD8-PerCP-Cy5.5 (eBioscience, Catalog No. 45-0081), CD4-FITC (eBioscience, Catalog No. 11-0042), CD44-PE (eBioscience, Catalog No. 12-0441) and CD62L-APC (eBioscience, Catalog No. 17-0621) antibodies and analyzed by FCM. All data were processed using the flow cytometry analysis software (FlowJo, version 10).

Multiple immunohistochemistry (mIHC) assay. mIHC was performed by using 5/4-Color Multiple IHC Kit (Absin Bioscience Inc., Shanghai, China). Tumor tissues were harvested from the mice. The tumor sections were cut with a cryotome, mounted on slides and labeled after embedded and fixed with different primary antibodies: Ki67 (Cell Signaling Technology, Catalog No. 9449), CD45 (Cell Signaling Technology, Catalog No. 70257), Ly6G (Cell Signaling Technology, Catalog No. 87048), CD11b (Abcam, Catalog No. ab184308), CD206 (Proteintech, Catalog No. 60143-1-Ig) and CD8 (Proteintech, Catalog No. 66868-1-Ig) and added the corresponding secondary antibodies following the manufacturer's instructions. Nuclei was then counterstained for DAPI. The images were analyzed by Halo software (V.3.1).

Immunofluorescence staining. Tumors were harvested from the mice and snap-frozen in optimum cutting temperature. The tumor sections were cut with a cryotome and mounted on slides. Sections were stained with different primary antibodies: Anti-p110γ antibody (Cell Signaling Technology, Catalog No. 4252) and anti-PD-L1 antibody (Biolengend, Catalog No. 124311) following the manufacturer's instructions. The slides were analyzed by a confocal microscope (Pannoramic MIDI, 3Dhistech).

Western blotting. Equal amounts of protein were mixed with an equal volume of 2 × Laemmli buffer and heated at 95 °C for 5 min. Afterward, four samples were loaded into the well of 8% SDS-PAGE gel and ran at 90 V for 60 minutes. After protein transformation, anti-p110γ antibody at a 1:1,000 dilution (Cell Signaling Technology, Catalog No. 4252) was used as primary antibody and incubated overnight at 4 °C. The secondary antibody was used for these blots at room temperature for 1 hour.

Statistical analysis. Data are shown as means ± standard deviation (SD), as indicated. Two groups were compared by Student's *t* test (two-sided). Tukey's post hoc tests and one-way analysis of variance (ANOVA) were performed for more than two group's comparisons. Statistical differences in survival were plotted using a Kaplan-Meier curve and assessed by a Log-Rank (Mantel-Cox) test. Statistical analysis was performed with GraphPad Prism 8, n.s., not significant, *$p < 0.05$, **$p < 0.01$, ***$p < 0.001$.

B. Supplementary figures

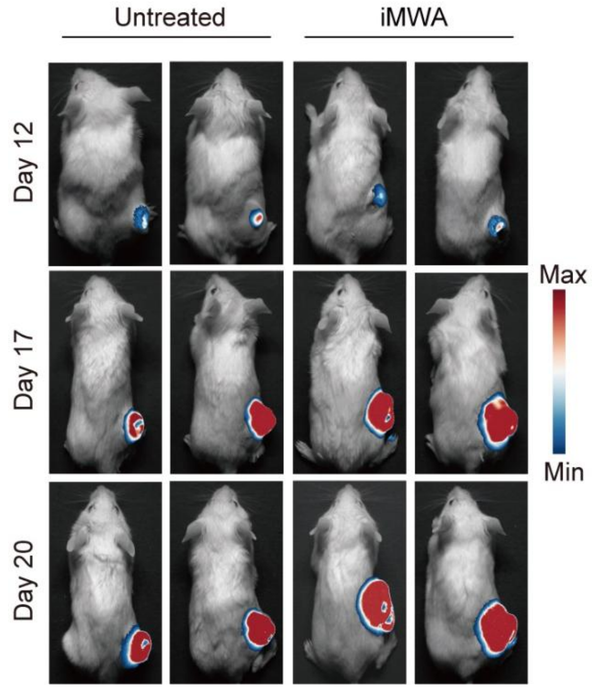

Figure S1. Representative *in vivo* bioluminescence images of mice post inadequate microwave ablation (iMWA).

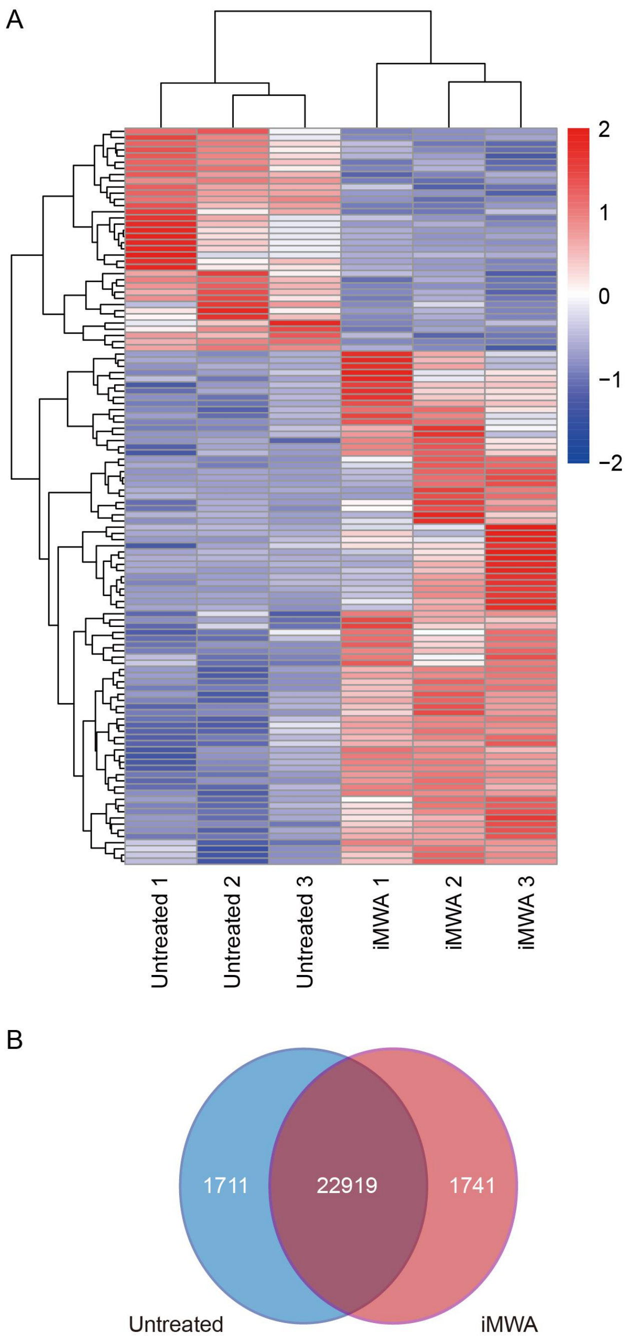

Figure S2. (A) Heatmap revealing differentially expressed genes and (B) venn diagram displaying the number of intersecting genes between the untreated and inadequate microwave ablation (iMWA)-treated tumors. Tumors were harvested at day 3 after iMWA (n = 3). Blue and red colors indicate downregulated and upregulated genes, respectively.

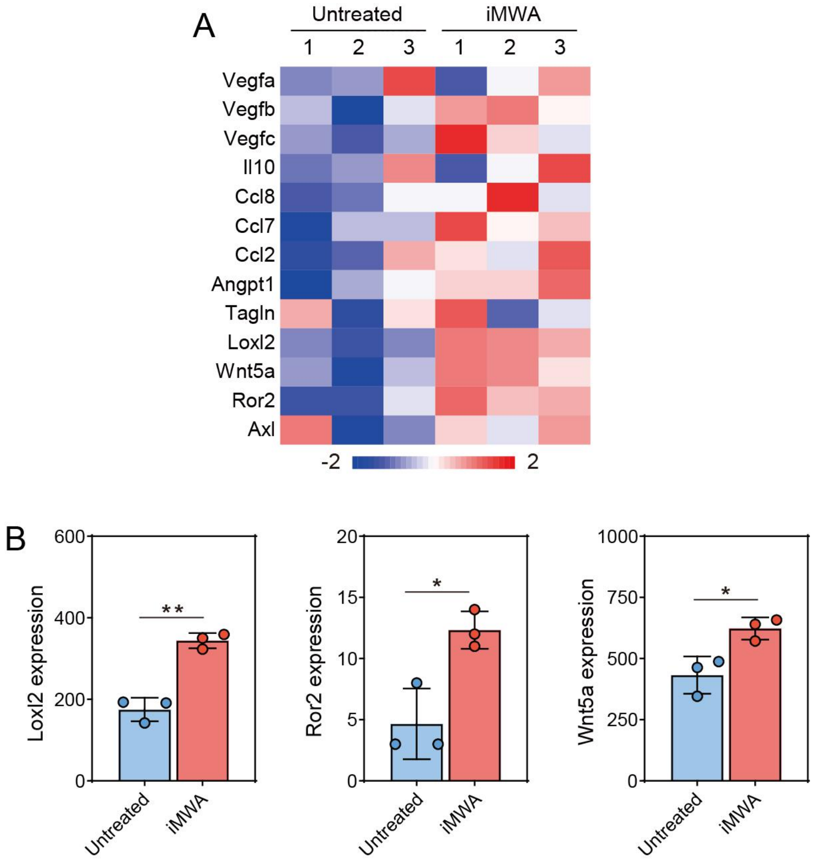

Figure S3. Heatmap of genes which are highly correlated with immune checkpoint blocking (ICB) resistance in clinic. (A) Heatmap and (B) quantification of genes which are highly correlated with ICB resistance in the untreated and residual tumors three days post ablation. Data are presented as means ± SD (n = 3). Statistical significance was calculated by Student's *t* test. *$p < 0.05$, **$p < 0.01$.

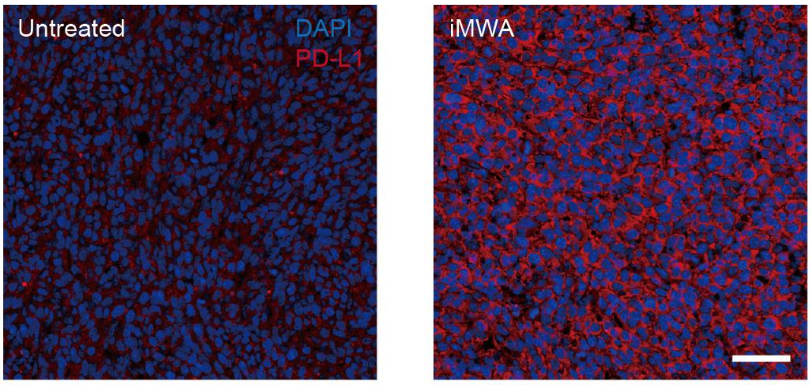

Figure S4. Representative immunofluorescence images of programmed death-ligand 1 (PD-L1) in untreated and residual tumor at 3 days after inadequate microwave ablation (iMWA). Scale bar: 50 μm.

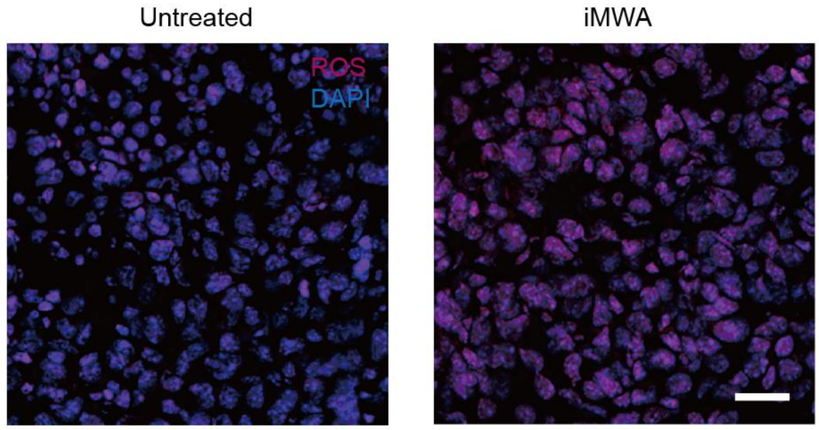

Figure S5. ROS in tumors before and after inadequate microwave ablation (iMWA) collected from mice were evaluated using the CellROX deep red reagent by confocal fluorescence. Scale bar: 10 μm.

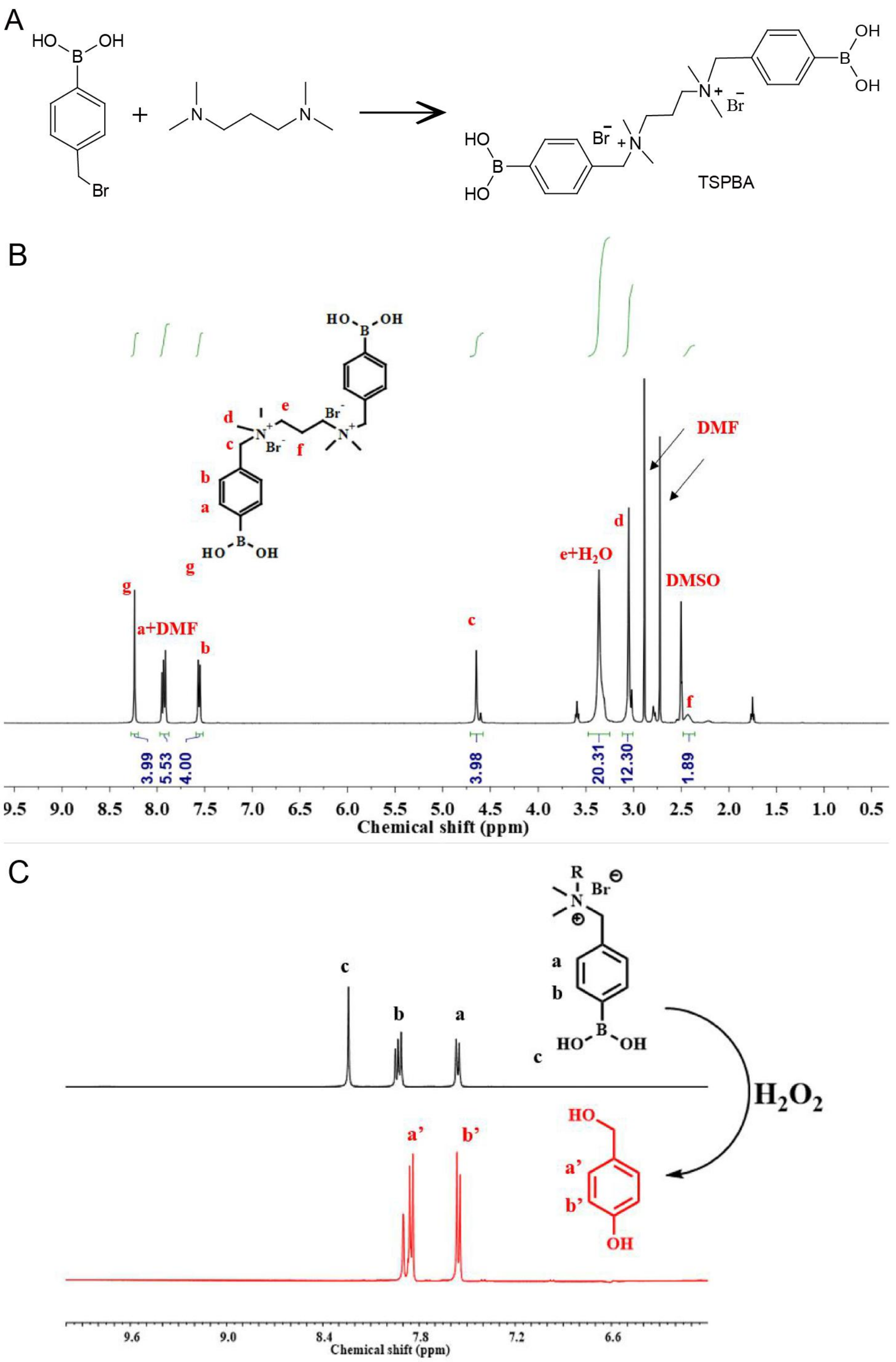

Figure S6. (A) Synthesis route of N^1-(4-boronobenzyl)-N^3-(4-boronophenyl)-N^1,N^1,N^3,N^3-tetramethylpropane-1,3-diaminium (TSPBA) and ^{1}H-NMR spectra of TSPBA linker in (B) d-DMSO and (C) D_2O with 1 mM H_2O_2.

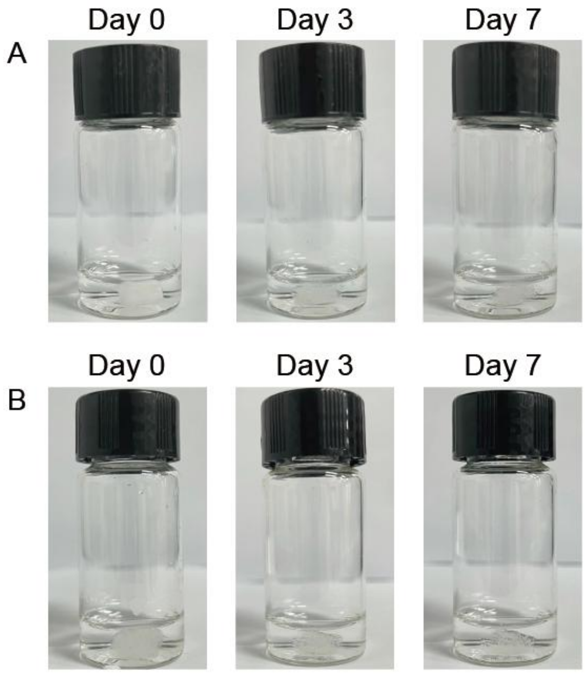

Figure S7. Degradation of ROS-sensitive hydrogel in PBS containing (A) 0.5 mM H_2O_2 and (B) 0.25 mM H_2O_2.

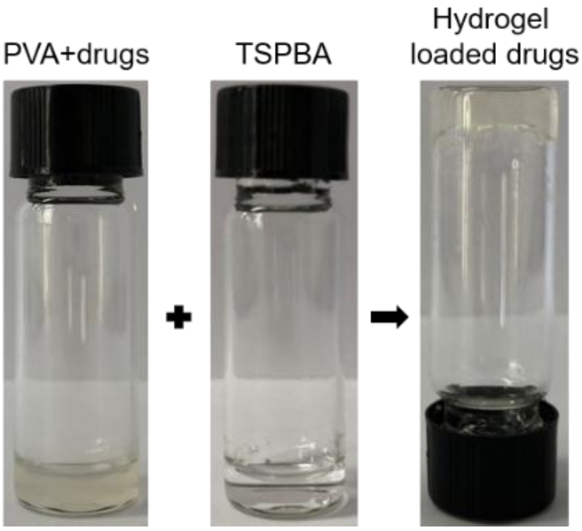

Figure S8. Pictures of the hydrogel's formation with the combining PVA (containing IPI549 and anti-programmed death-ligand 1 blocking antibody (aPDL1)) and TSPBA.

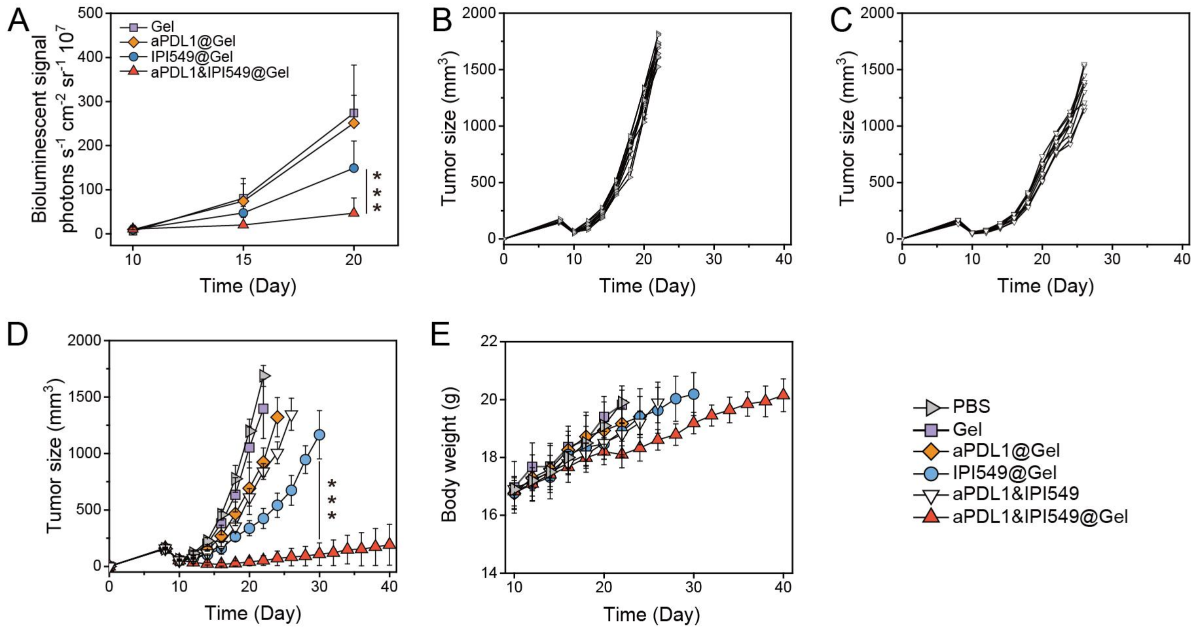

Figure S9. (A) The analysis of bioluminescence signals in four groups. (B and C) Individual and (D) average residual tumor growth kinetics in different groups. (E) Average body weights of mice in various therapeutics. Data are presented as means ± SD (n = 9-10). Statistical significance was calculated by one-way ANOVA with a Tukey post-hoc test. ***$p < 0.001$.

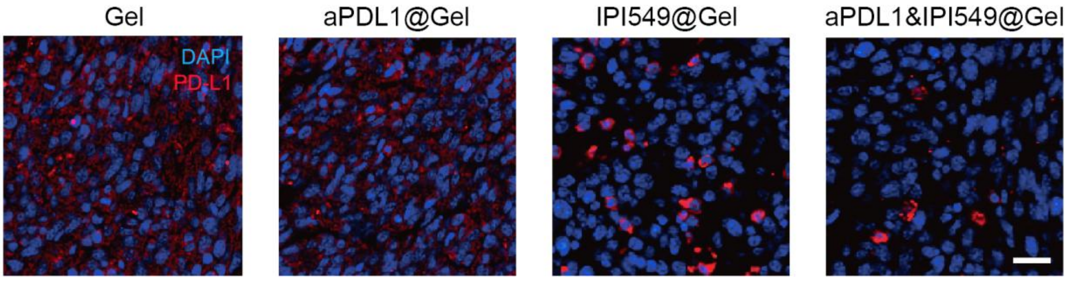

Figure S10. Representative immunofluorescence images of programmed death-ligand 1 (PD-L1) after various treatments. Scale bar: 20 μm.

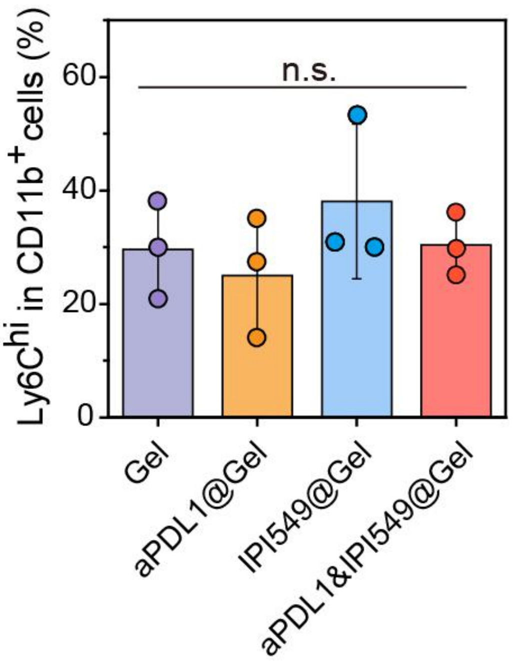

Figure S11. Flow cytometric quantification of monocytic myeloid derived suppressor cells (mMDSCs; CD11b$^+$Ly6C^{hi}) gating on CD45$^+$ cells after various treatments. Data are presented as means ± SD (n = 3). Statistical significance was calculated by one-way ANOVA with a Tukey post-hoc test. n.s., not significant.

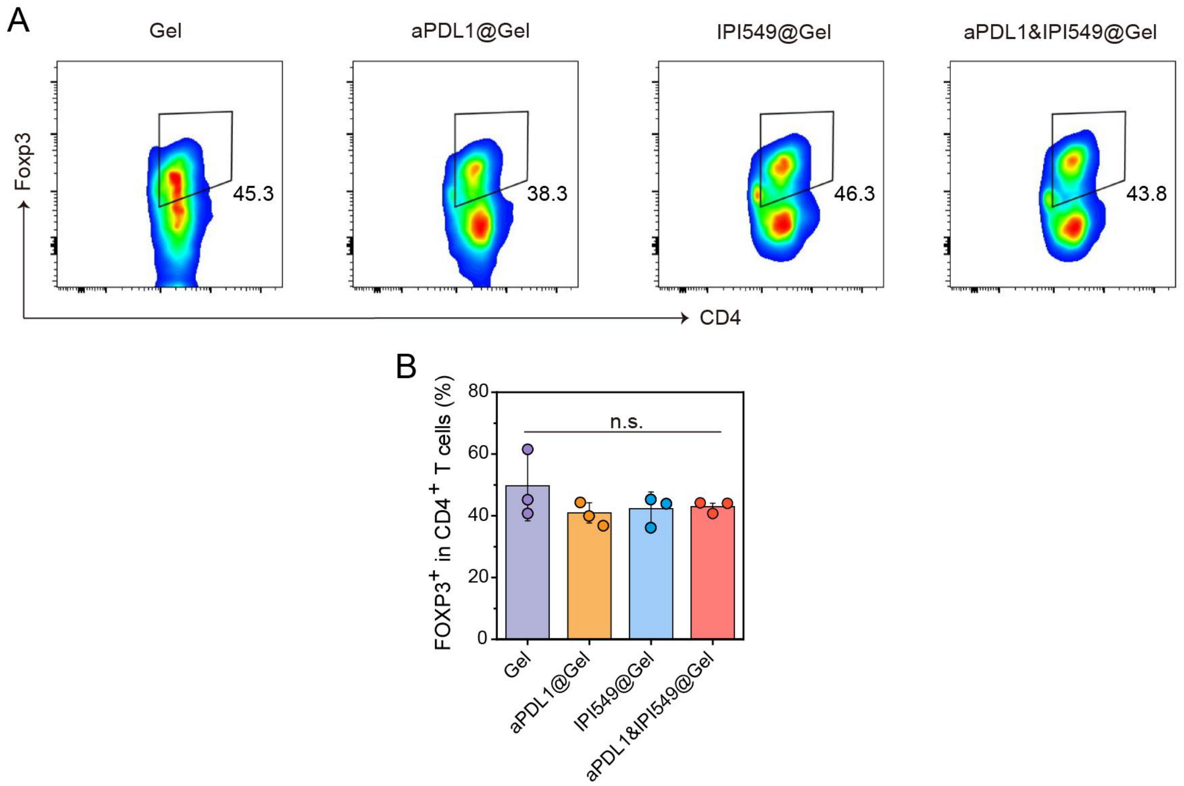

Figure S12. Representative flow cytometric (A) analysis and (B) quantification of $CD4^{+}Foxp3^{+}$ T cells gating on $CD3^{+}$ cells after various treatments. Data are presented as means ± SD (n = 3). Statistical significance was calculated by one-way ANOVA with a Tukey post-hoc test. n.s., not significant.

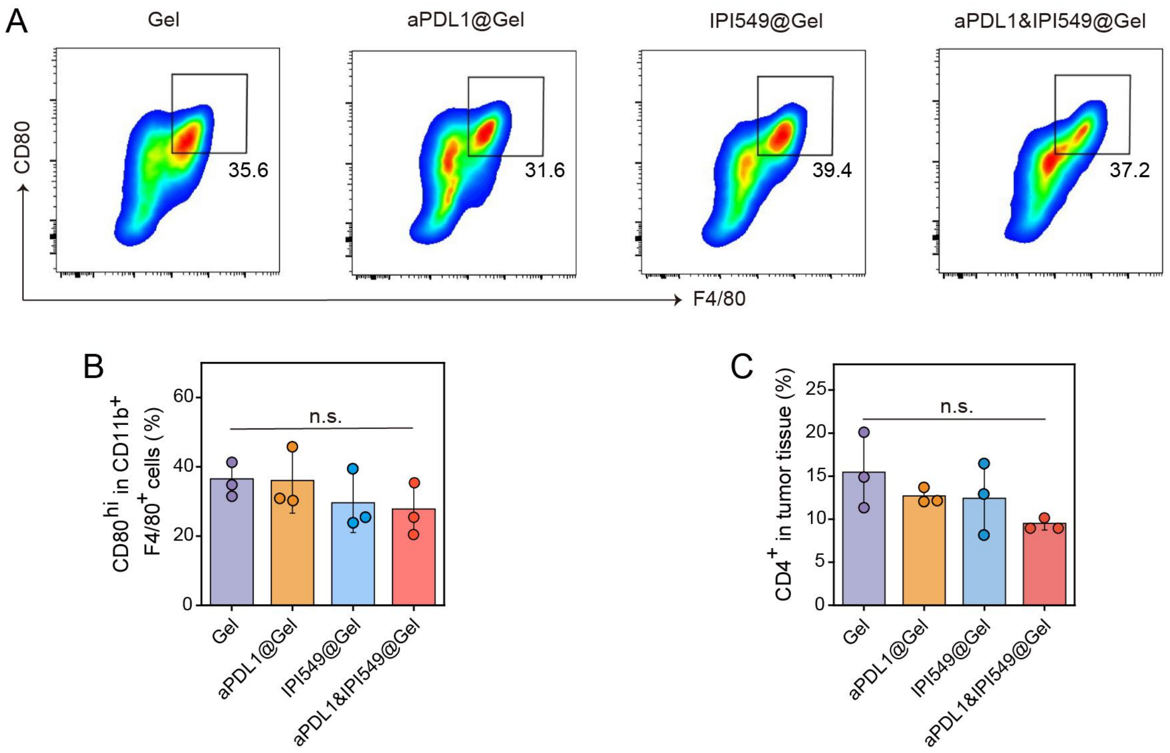

Figure S13. Representative flow cytometric (A) analysis and (B) quantification of TAMs-M1(CD80^{hi}) in CD11b^{+}F4/80^{+} cell population and (C) quantification of CD4^{+} T cells gating on CD45^{+}CD3^{+} cells after various treatments. Data are presented as means ± SD (n = 3). Statistical significance was calculated by one-way ANOVA with a Tukey post-hoc test. n.s., not significant. TAMs, tumor-associated macrophages; M1, M1-like macrophage.

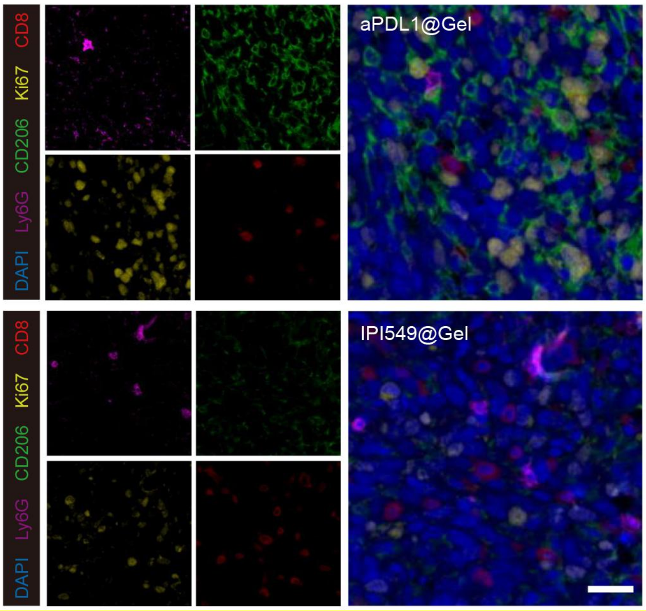

Figure S14. Representative immunofluorescence images of residual tumors displaying $CD8^+$ T cell, Ki67, CD206 and Ly6G infiltration in aPDL1@Gel and IPI549@Gel group. Scale bar: 20 μm. aPDL1, anti-programmed death-ligand 1 blocking antibody.

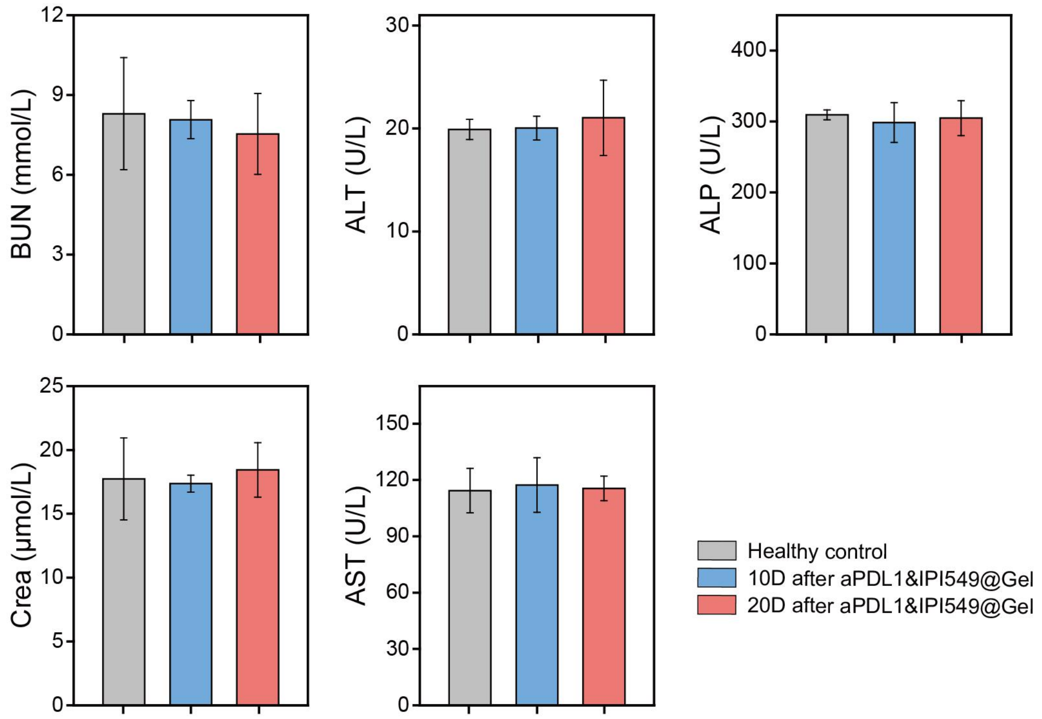

Figure S15. Serum biochemistry data. Female BALB/c mice were sacrificed at day 10 and day 20 after local injection of aPDL1&IPI549@Gel. Healthy mice were identified as controls. Serum biochemistry data including blood urea nitrogen (BUN), alanine aminotransferase (ALT), alkaline phosphatase (ALP), creatinine (Crea) and aspartate aminotransferase (AST) were measured. Data are presented as means ± SD (n = 3). aPDL1, anti-programmed death-ligand 1 blocking antibody.

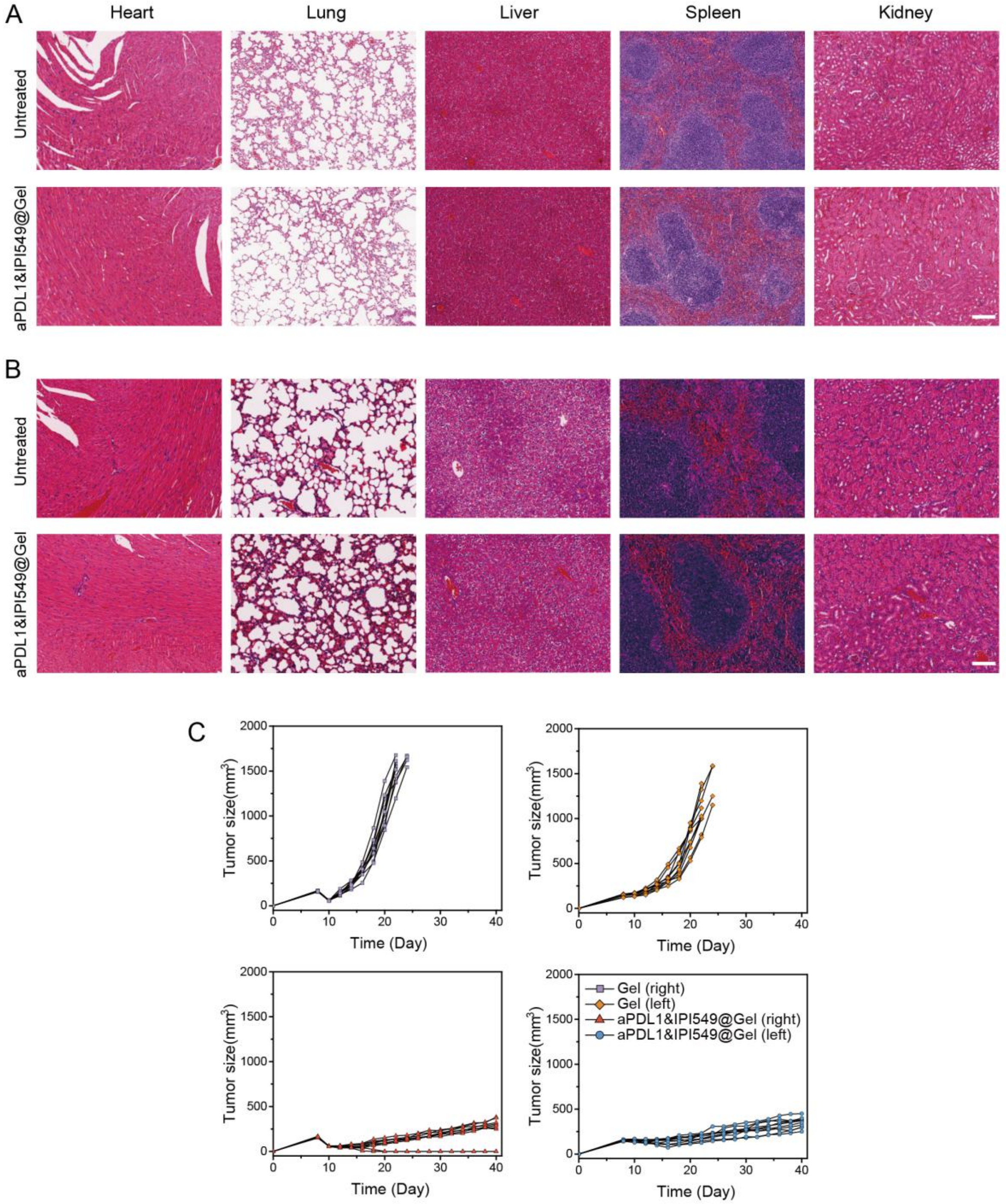

Figure S16. (A and B) Biological safety of aPDL1&IPI549@Gel *in vivo* and (C) tumor growth kinetics of mice treated with Gel or aPDL1&IPI549@Gel. Hematoxylin and eosin (H&E) staining sections of the major organs collected from healthy and aPDL1&IPI549@Gel treated mice at day 10 (A) and day 20 (B). Scale bar: 250 µm. aPDL1, anti-programmed death-ligand 1 blocking antibody.

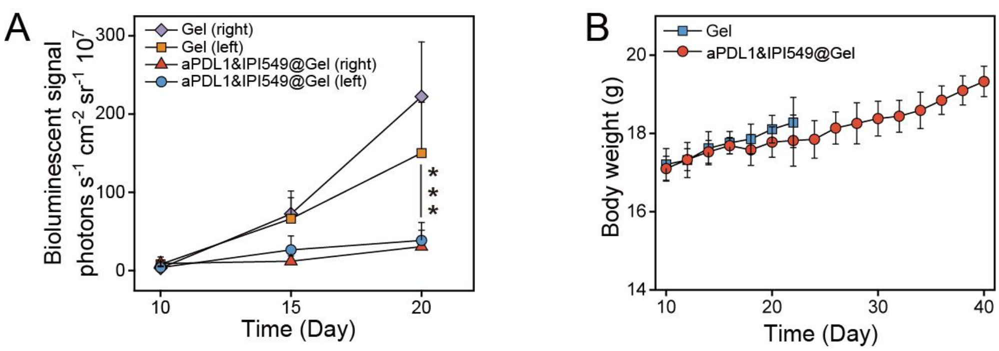

Figure S17. (A) The analysis of bioluminescence signals of tumor in both sides. (B) Body weights of mice after treated with Gel or aPDL1&IPI549@Gel. Data are shown as means ± SD (n = 10). Statistical significance was calculated by one-way ANOVA with a Tukey post-hoc test. ***$p < 0.001$. aPDL1, anti-programmed death-ligand 1 blocking antibody.

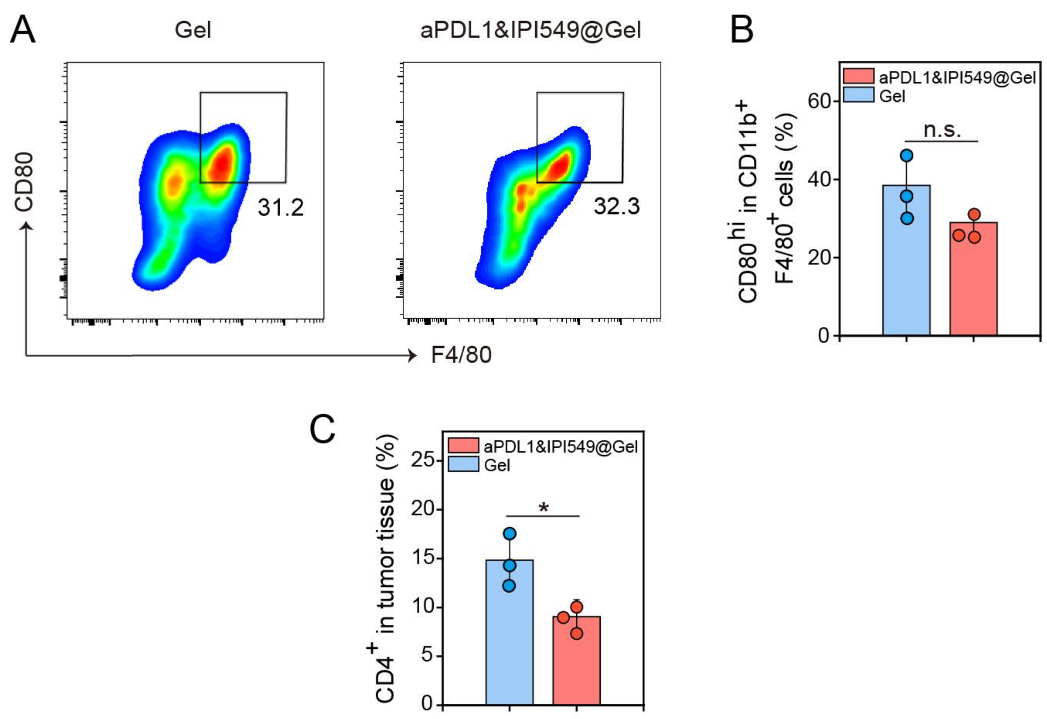

Figure S18. Representative flow cytometric (A) analysis and (B) quantification of TAMs-M1 ($CD80^{hi}$) gating on $CD11b^{+}F4/80^{+}$ cells and (C) quantification of $CD4^{+}$ T cells gating on $CD45^{+}CD3^{+}$ cells after different treatments. Data are presented as means ± SD (n = 3). Statistical significance was calculated by Student's *t* test. n.s., not significant. $^{*}p < 0.05$. TAMs, tumor-associated macrophages; M1, M1-like macrophage.

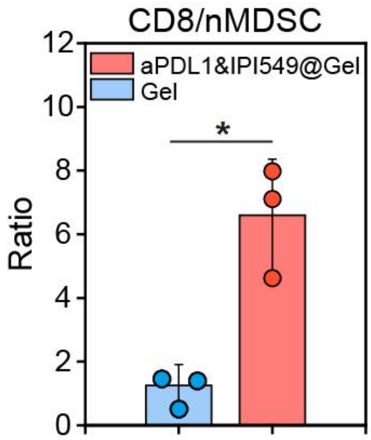

Figure S19. Quantification by flow cytometry of CD8/nMDSC ratio. Data are presented as means ± SD (n = 3). Statistical significance was calculated by Student's *t* test. *$p < 0.05$. nMDSC, neutrophilic myeloid derived suppressor cell.

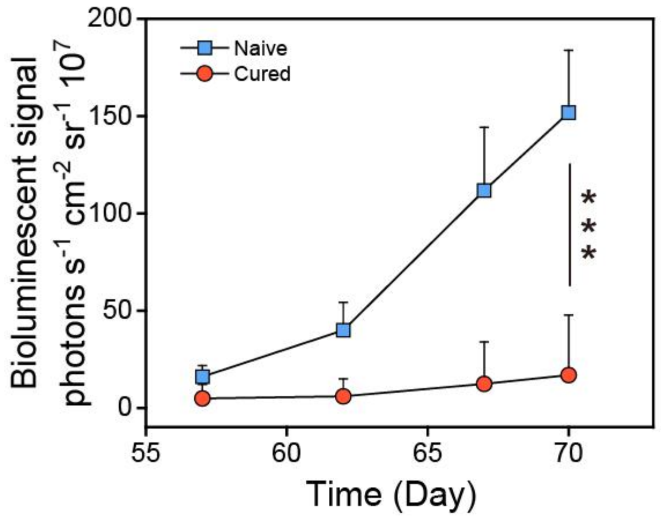

Figure S20. The analysis of bioluminescence signals of the naive and cured mice. Data are presented as means ± SD (n = 4). Statistical significance was calculated by Student's *t* test. ***$p < 0.001$.

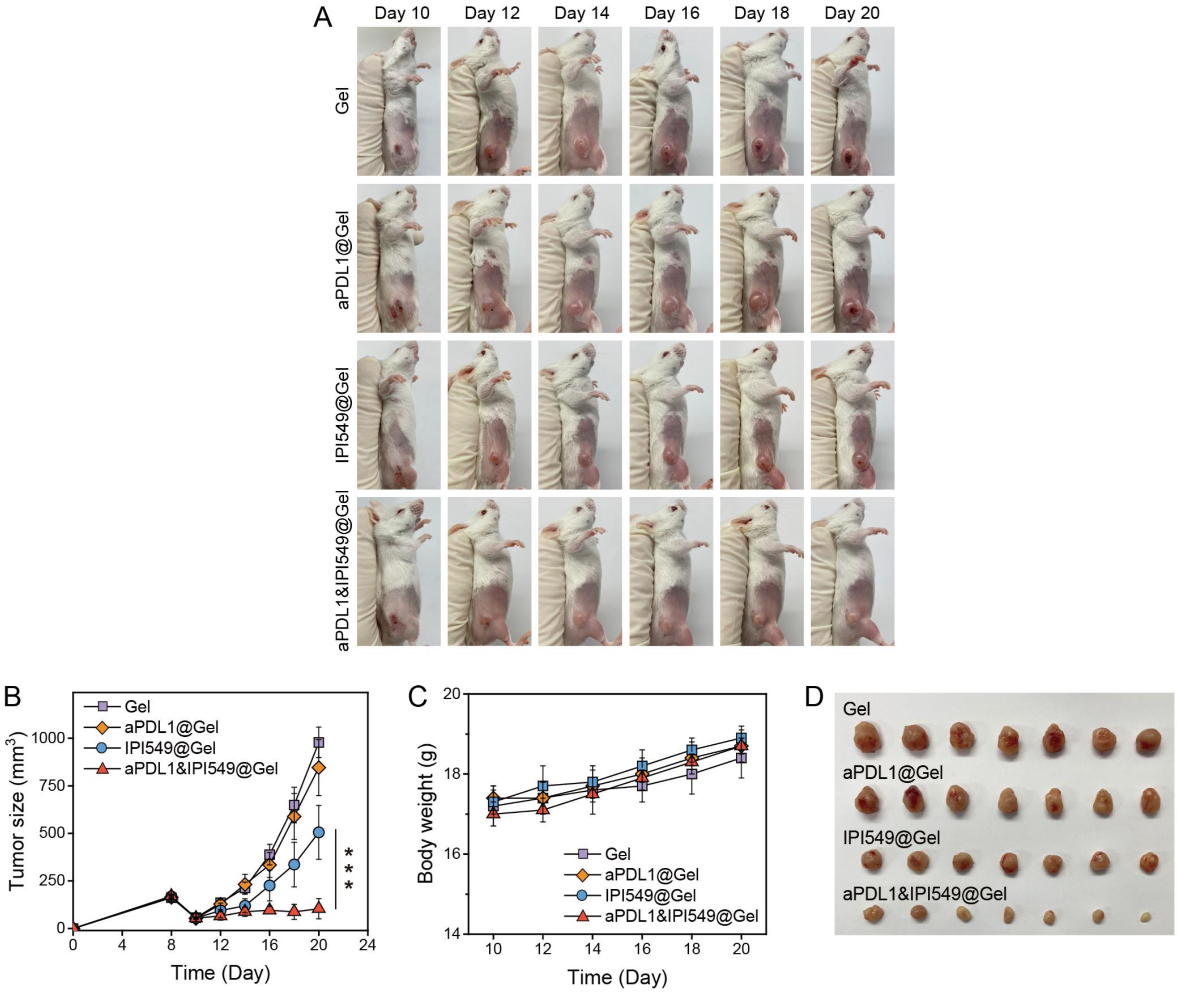

Figure S21. (A) Photographs of 4T1 tumor-bearing mice at different periods after various treatment. (B) Individual residual tumor growth kinetics in different groups. (C) Average body weights of mice in various therapeutics. (D) Photographs of excised residual tumors on day 20 after various treatment. Data are presented as means ± SD (n = 7). Statistical significance was calculated by one-way ANOVA with a Tukey post-hoc test. $^{***}p < 0.001$.
